# Rapid, efficient and activation-neutral gene editing of polyclonal primary human resting CD4^+^ T cells allows complex functional analyses

**DOI:** 10.1038/s41592-021-01328-8

**Published:** 2021-12-23

**Authors:** Manuel Albanese, Adrian Ruhle, Jennifer Mittermaier, Ernesto Mejías-Pérez, Madeleine Gapp, Andreas Linder, Niklas A. Schmacke, Katharina Hofmann, Alexandru A. Hennrich, David N. Levy, Andreas Humpe, Karl-Klaus Conzelmann, Veit Hornung, Oliver T. Fackler, Oliver T. Keppler

**Affiliations:** 1 Max von Pettenkofer Institute and Gene Center, Virology, National Reference Center for Retroviruses, Faculty of Medicine, LMU München, Munich, Germany; 2 Gene Center and Department of Biochemistry, LMU München, Munich, Germany; 3 Department of Medicine II, University Hospital, LMU München, Munich, Germany; 4 Department of Molecular Pathobiology, New York University College of Dentistry, New York, NY USA; 5 Department of Transfusion Medicine, Cell Therapeutics, and Hemostaseology, Department of Anesthesiology, University Hospital Munich, Munich, Germany; 6 German Centre for Infection Research (DZIF), Partner Site Munich, Munich, Germany; 7 Department of Infectious Diseases, Integrative Virology, University Hospital Heidelberg, Heidelberg, Germany; 8 German Centre for Infection Research (DZIF), Partner Site Heidelberg, Heidelberg, Germany; 9 Present Address: Istituto Nazionale di Genetica Molecolare, INGM, “Romeo ed Enrica Invernizzi”, Milan, Italy

**Keywords:** Gene regulation, Infectious diseases, Adaptive immunity, Virology, Lymphocytes

## Abstract

CD4^+^ T cells are central mediators of adaptive and innate immune responses and constitute a major reservoir for human immunodeficiency virus (HIV) in vivo. Detailed investigations of resting human CD4^+^ T cells have been precluded by the absence of efficient approaches for genetic manipulation limiting our understanding of HIV replication and restricting efforts to find a cure. Here we report a method for rapid, efficient, activation-neutral gene editing of resting, polyclonal human CD4^+^ T cells using optimized cell cultivation and nucleofection conditions of Cas9–guide RNA ribonucleoprotein complexes. Up to six genes, including HIV dependency and restriction factors, were knocked out individually or simultaneously and functionally characterized. Moreover, we demonstrate the knock in of double-stranded DNA donor templates into different endogenous loci, enabling the study of the physiological interplay of cellular and viral components at single-cell resolution. Together, this technique allows improved molecular and functional characterizations of HIV biology and general immune functions in resting CD4^+^ T cells.

## Abstract (web summary)

A nucleofection-based method to enable efficient gene editing of primary human resting CD4^+^ T cells.

## Main

While HIV-1 readily infects and replicates in activated CD4^+^ T cells, resting CD4^+^ T cells are highly resistant to productive HIV infection^1^. The use of cells derived from patients with Aicardi–Goutières syndrome carrying a defect in the *SAMHD1* gene^2,3^ as well as the use of lentiviral Vpx proteins to increase the susceptibility of noncycling cells to HIV-1 infection has led to the identification of SAMHD1 as a critical barrier for reverse transcription of incoming viral genomes^3–6^. Subsequent studies have hinted at a number of additional replication blocks in resting CD4^+^ T cells^7,8^. However, gaining more comprehensive mechanistic insight into the interplay of HIV with these abundant primary target cells for infection, replication and latency, requires new gene editing technology and optimized cultivation protocols.

Standard transfection and transduction protocols have failed in this cell type and are mainly used on CD4^+^ T cells that are first activated and then allowed to return to a phenotypically resting state. The analysis of these ‘post-activation’ resting CD4^+^ T cells, which had been manipulated by either short-hairpin RNA (shRNA)-encoding lentiviral vectors or nucleofection with short-interfering RNAs (siRNAs) or guide RNA (gRNA)–Cas9 complexes^3,9,10^ to target a single cellular factor, has provided some functional insights. However, this approach is generally inefficient, labor-intensive and does not faithfully reflect the resting state of naive human CD4^+^ T cells. In the current study, we aimed at developing an efficient, rapid, nonviral, plasmid and selection-free approach to introduce complex genetic alterations in resting human CD4^+^ T cells.

## Results

### Protocol for a highly efficient, polyclonal, multi-gene knockout in primary human resting CD4^+^ T cells

Cas9–gRNA ribonucleoproteins (RNPs) have been successfully employed for gene editing of activated T cells^10–14^ as well as the establishment of single-gene knockouts (KOs), albeit at limited efficacy and accompanied by marked cytotoxicity in resting T cells, thus precluding functional readouts beyond day 3 of cultivation^12^. We attempted to optimize and extend this approach to resting CD4^+^ T cells.

**Fig. 1:**
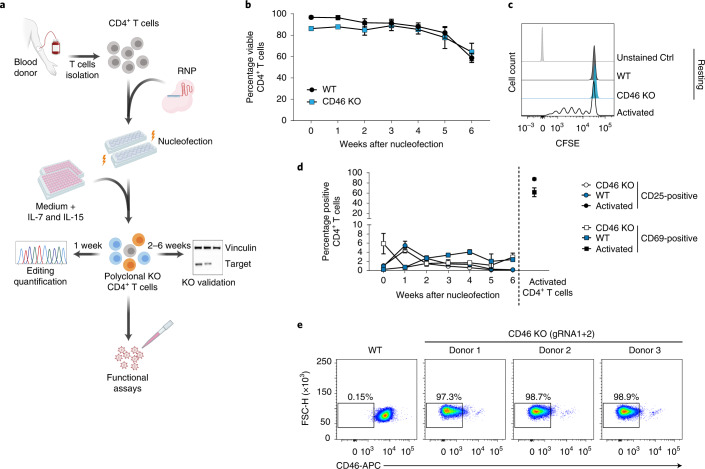
Highly efficient KO generation in primary human resting CD4^+^ T cells. **a**, Schematic overview of the pipeline to establish polyclonal KOs in human resting CD4^+^ T cells. **b**, Viability of CD46 KO or untreated WT resting CD4^+^ T cells kept in culture for up to 6 weeks. Cell viability was assessed at the indicated time points by flow cytometry. Mean ± s.e.m. are shown (*n* = 3). **c**, T-cell proliferation assay. CFSE-labeled CD46 KO and WT CD4^+^ T cells analyzed by flow cytometry 1 week after nucleofection. Anti-CD3/CD28 antibody-activated CD4^+^ T cells (activated) served as positive control (Ctrl). One representative experiment is shown (*n* = 3). **d**, WT and CD46 KO resting CD4^+^ T cells were analyzed by flow cytometry for expression of T-cell activation markers CD25 and CD69. Mean ± s.e.m. are shown (*n* = 3). **e**, Resting CD4^+^ T cells (WT or CD46 KO) were analyzed for cell surface expression of CD46 2 weeks after nucleofection. Shown are dot plots of flow cytometric analyses for cells from three individual donors and WT cells from one of these donors. Fig. 1a created with BioRender.com. Source data

**Extended Data Fig. 1:**
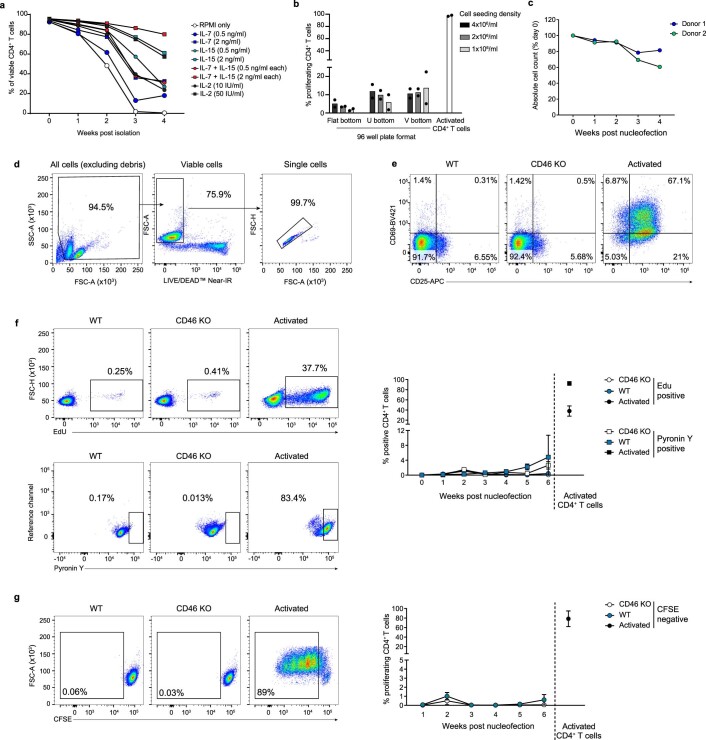
Basic culture conditions to keep human CD4^+^ T cells viable and resting. **a**, Combined IL-7/IL-15 treatment is optimal for resting CD4^+^ T cells. Primary CD4^+^ T cells were enriched from PBMCs from healthy donors using the EasySep Rosette Human CD4^+^ T Cell enrichment kit (negative selection). Isolated cells were kept in culture for 4 weeks with the addition of the indicated interleukins to the culture medium. The interleukin supplement was refreshed twice a week. Once a week an aliquot of cells was analyzed by staining with LIVE/DEAD^TM^ Fixable Near-IR Dead Cell Stain to assess the culture’s viability. Means are shown (n = 2). **b**, Resting CD4^+^ T cells were labeled with CFSE, seeded at the indicated cell concentrations and plate formats (flat bottom, U bottom, V bottom), and maintained in culture with medium containing IL-7/IL-15 (both 2 ng/ml). Two weeks later, cell proliferation was assessed by flow cytometry. Means are shown (n = 2). Anti-CD3/CD28 mAb-activated CD4^+^ T cells served as positive control. **c**, Once a week over a period of four weeks WT cells were analyzed by flow cytometry using BD Trucount™ Tubes to assess absolute cell counts. Data from two independent donors are shown. **d**, Gating strategy to assess cell viability by staining with LIVE/DEAD^TM^ Fixable Near-IR Dead Cell Stain. **e-g**, RNP nucleofection per se does not alter cell’s activation state. Resting CD4^+^ T cells were nucleofected with RNPs specific for *CD46* (gRNA1 + 2), and CD46 KO cells and WT cells were maintained in culture with medium containing IL-7/IL-15 (both 2 ng/ml) and (**e**) analyzed two weeks later for expression of T cell activation markers CD25 and CD69. Anti-CD3/CD28 mAb-activated CD4^+^ T cells served as positive control. **f,g**, Once a week over a period of six weeks, cells were also analyzed by flow cytometry for the following parameters: DNA synthesis by EdU incorporation assay (**f**, top panels), RNA content quantification by Pyronin Y staining (**f**, bottom panels), cell proliferation by CFSE staining (**g**). Left panels show one representative example at week 2 after nucleofection. Right panels are the summary of all results with means ± s.e.m. (n = 3). Note that EdU incorporation scores cell division only at the time point of analysis, while CSFE analysis tracks cell division events during the entire cultivation period. Typically, both markers stayed below 1% positivity in cultures for up to 6 weeks, consistent with a lack of proliferation. Source data

**Extended Data Fig. 2:**
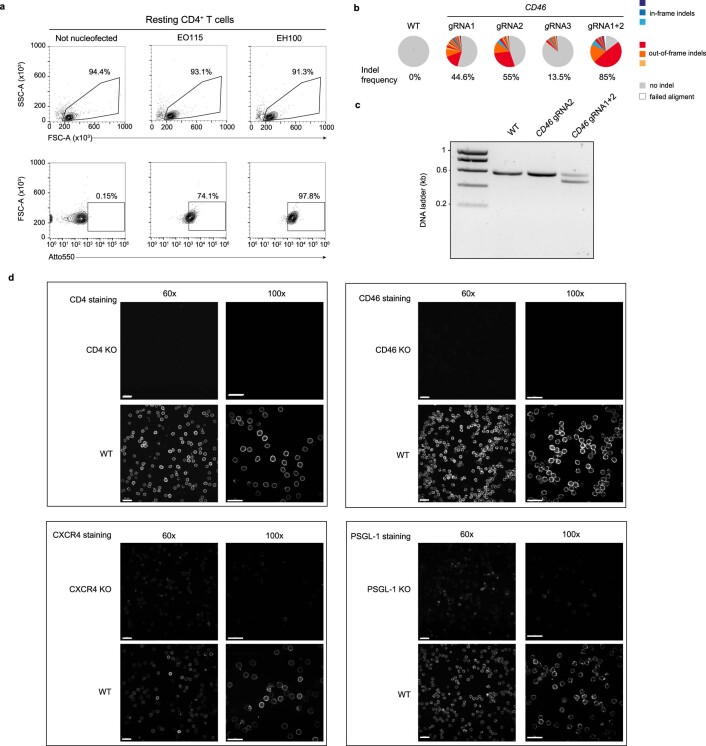
Optimized nucleofection allows RNP delivery into virtually every resting CD4^+^ T cell and results in efficient KO of single genes. **a**, RNPs were generated by mixing crispr RNA (crRNA) together with a fluorescently labeled tracrRNA (Atto550; IDT) in an equimolar ratio and incubation at 95 °C for 5 min. The temperature was then decreased slowly to reach room temperature (around 1 hour). Next, the gRNA complex was mixed together with CAS9 for 15 min at room temperature, at molar ratios of 1:2.5 (Cas9:gRNA). Cells were mixed with the RNP complex and nucleofected in P3 buffer using either nucleofection program EH100 or EO115 (the latter suggested by the manufacturer). 24 h after nucleofection, the positivity for the fluorescent Atto5 label was analyzed by flow cytometry on a BD LSRFortessa. Cells mixed with the RNP fluorescent complex, but not nucleofected, served as control. FACS plots of one out of two experiments is shown. **b**,**c** Resting CD4^+^ T cells were nucleofected with RNPs specific for *CD46* using either gRNA1, gRNA2, gRNA3 or a combination of gRNA1 + 2. One week later, cells were collected and lysed. A PCR specific for the *CD46* locus was performed. The product was either analyzed by deep sequencing on an Illumina Miseq (**b**) or separated on an agarose gel (**c**). One experiment is shown (n = 2). **d**, Qualitative evaluation of the different gene KOs in resting CD4^+^ T cells by immunostaining and confocal microscopy two weeks after nucleofection. One representative donor each out of three is shown. Scale bars: 20 µm.

As the viability of these cells, purified from the leukoreduction system chambers from peripheral blood of healthy donors, declines rapidly even in the presence of interleukin (IL)-2 (Extended Data Fig. 1a), we compared several IL supplements, cell densities and plate formats for long-term cultivation. Extending previous reports^15,16^, addition of low concentrations of human IL-7 and IL-15 to the culture medium significantly improved cell survival (Fig. 1a,b and Extended Data Fig. 1a–c) without inducing cell proliferation or expression of activation markers CD25 and CD69 (Fig. 1c,d and Extended Data Fig. 1d–g). In addition, a low starting cell density and flat-bottom culture plates facilitated the preservation of the resting state (Extended Data Fig. 1c). The use of an RNP containing a fluorescently labeled *trans*-activating crispr RNA (tracrRNA) delivered into freshly isolated resting CD4^+^ T cells identified 4D-Nucleofector protocol EH-100 as optimal, in line with findings by Seki and Rutz^12^, achieving over 97% RNP delivery (Extended Data Fig. 2a).

This resulted in a workflow (Fig. 1a and Methods) where resting CD4^+^ T cells are nucleofected once with RNPs and then cultivated under specific conditions for up to 6 weeks until assessment of gene editing and functional characterization. To establish the KO efficacy by this protocol, we first targeted *CD46*, which encodes a single-span transmembrane cell surface receptor and functions as an important regulator of the complement system^17^. CD46 is homogenously expressed on resting CD4^+^ T cells and other hematopoietic cells^18^. Nucleofection of resting CD4^+^ T cells with RNPs containing *CD46*-targeting gRNAs did not affect cell viability compared to untreated cells (wild-type (WT)) with over 80% viable cells for up to 5 weeks (Fig. 1b). Based on the determination of absolute cell numbers (Extended Data Fig. 1c) we established that nucleofection of approximately 2.5-fold more cells than required for downstream functional characterization represents a reliable workflow.

**Extended Data Fig. 3:**
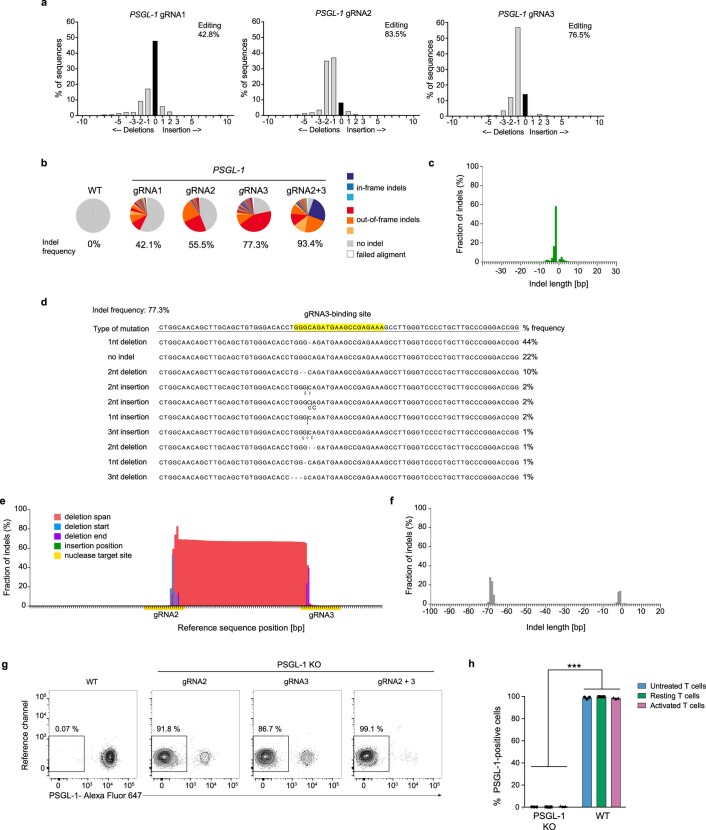
PSGL-1 KO in resting CD4^+^ T cells. Cells were nucleofected with RNPs specific for *PSGL-1/SELPLG* using either gRNA1, gRNA2, gRNA3 or a combination of gRNA2 + 3. One week after nucleofection, cells were collected and lysed. A PCR specific for the *PSGL-1* locus was performed and analyzed by TIDE analysis (**a**) or Illumina Miseq (**b**). **c**, Analysis of the indel size distribution from the PSGL-1 KO generated with gRNA3 in the *PSGL-1* locus. **d**, Sequence alignment of the different indels found in **c**. **e-f**, Analysis of the indels (e) and their size distribution (**f**) from the PSGL-1 KO generated with gRNA2 + 3 in the *PSGL-1* locus. One representative experiment is shown (n = 3). Outknocker (http://www.outknocker.org/) was used for sequence analysis. **g**, PSGL-1 surface expression two weeks after nucleofection of the indicated gRNAs. Density plots of flow cytometric analysis of viable, resting (CD25^−^/CD69^−^) CD4^+^ T cells of one representative experiment are shown (n = 3). **h**, Resting CD4^+^ T cells were nucleofected with *PSGL-1*-targeting gRNA2+ 3. Subsequently, T cells were either cultivated in IL-7/IL-15 (both 2 ng/ml) or activated using anti-CD3/CD28 mAb and IL-2 (50 IU/ml), or maintained untreated. Interleukins were refreshed twice a week. Two weeks later, PSGL-1 expression was analyzed by flow cytometry. Means ± s.e.m. are shown (n = 3). Statistics indicate significance by two-ways ANOVA. P-values were corrected for multiple comparison (Tukey)(***P ≤ 0.001). Source data

Nucleofected cells maintained their resting phenotype as assessed by multiple established markers in comparison to activated CD4^+^ T-cell reference cultures (Fig. 1c,d and Extended Data Fig. 1e–g). Three different *CD46*-gRNAs, targeting the *CD46* locus, were tested and the gene editing efficiency was quantified by deep sequencing 1 week after nucleofection (Extended Data Fig. 2b). The combination of the two most efficient *CD46-*targeting gRNAs (gRNA1 and gRNA2) resulted in editing of *CD46* in 85% of cells (Extended Data Fig. 2b,c) and an almost complete loss (97.3 to 98.9%) of CD46 cell surface expression 2 weeks after nucleofection as assessed by flow cytometry (Fig. 1e) and confocal microscopy (Extended Data Fig. 2d). Similar results were obtained for the cell surface receptor P-selectin glycoprotein ligand-1 (PSGL-1, CD162). Again, the combination of two *PSGL-1/SELPLG*-targeting gRNAs (gRNA2 + 3) resulted in the best editing efficacy (93.4%) (Extended Data Fig. 3a–f) and exposure of the PSGL-1 receptor on the surface of resting CD4^+^ T cells became almost undetectable 2 weeks after nucleofection (Extended Data Fig. 3g). Thereafter, combinations of two or three pre-tested gRNAs were successfully used for all genes targeted. Of note, the KO efficiency per se was independent of the cultivation and activation conditions following nucleofection (Extended Data Fig. 3h).

**Fig. 2:**
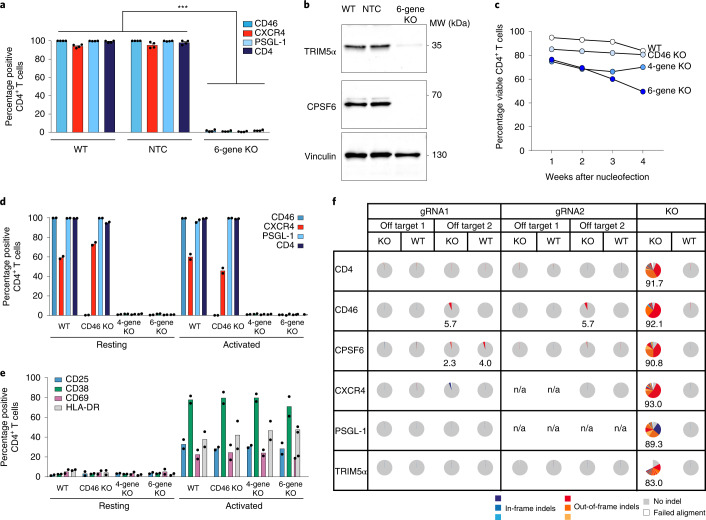
Polyclonal KO of up to six genes in primary human resting CD4^+^ T cells. **a**, Simultaneous, polyclonal six-gene KO following a single RNP nucleofection. Expression of surface receptors was quantified by flow cytometry after 2 weeks. Mean ± s.e.m. are shown (*n* = 4). Statistics indicate significance by two-way analysis of variance (ANOVA). *P* values were corrected for multiple comparison (Tukey). ***P ≤ 0.001. **b**, Immunoblot analysis of cell lysates from the experiment shown in **a** 25 d after nucleofection. All targets were validated on the same membrane by re-probing. One representative experiment is shown (*n* = 2). **c**, Viability of cells with *CD46*-single KO, four-gene KO (*CD46, CD4, CXCR4* and *PSGL-1*) or six-gene KO (as in **a**) were analyzed. WT cells served as control. Means are shown (*n* = 2). **d**,**e**, Resting cells were nucleofected, cultivated for 2 weeks and then either activated or not for one additional week before analyzing expression of the indicated surface receptors (**d**) and T-cell activation markers CD25, CD69, CD38 and HLA-DR (**e**) by flow cytometry. Means are shown (*n* = 2). **f**, Off-target analysis in resting CD4^+^ T cells following six-gene KO (as in **a**). Specific primers were designed to target the top two off-target coding loci predicted for each gRNA used (Extended Data Table 1). One week after nucleofection, cells were collected and lysed. A PCR specific for each off-target site was performed and analyzed by Illumina MiSeq. WT cells from the same donors served as control. Results from one out of two donors are shown. Percentages of indel frequencies are shown if >0.5%. NA, not available. Source data

**Extended Data Fig. 4:**
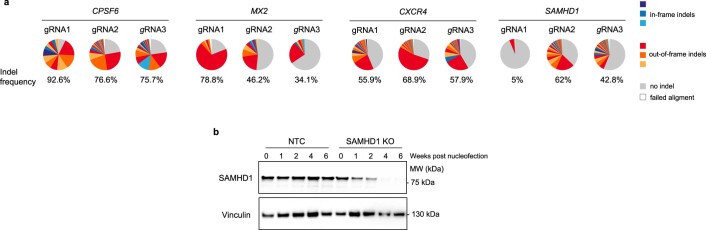
Characterization of different KOs in resting CD4^+^ T cells. **a**, Evaluation of KO efficiency in polyclonal resting CD4^+^ T cells of single gRNAs for genes studied in functional assays. Cells were nucleofected with RNPs specific for the indicated genes, and the KO efficiency was evaluated by Illumina Miseq one week after nucleofection. Representative examples for each gRNA are shown. Together with TIDE analysis, this method was used to validate individual gRNAs. Depending on their efficiency, a combination of the two most potent gRNAs was used to obtain >95% KO efficiency, unless indicated otherwise. **b**, SAMHD1 decay in RNP-nucleofected cultivated for up to 6 weeks. Cells were nucleofected with either *SAMHD1*-gRNA2 + 3 or NTC and cultivated for the indicated period of time. Shown are immunoblots for either SAMHD1 or vinculin (loading control). One representative experiment is shown (n = 4).

Next, we wanted to test the applicability of this protocol for a multi-gene KO approach. As such, we targeted the expression of five genes with roles in HIV-1 infection (*CD4*, *CXCR4*, *PSGL-1*, *TRIM5α* and *CPSF6*) as well as *CD46*, in part using gRNAs pre-tested for each target for the efficiency of editing (Extended Data Fig. 4a) and protein loss was assessed in part by confocal microscopy (Extended Data Fig. 2d). The use of 12 different gRNAs in a single RNP complex nucleofection resulted in a reduction of the expression of all six proteins to almost undetectable levels at the cell surface as determined by flow cytometry 2 weeks after nucleofection for CD46, CXCR4, PSGL-1 and CD4 (Fig. 2a) or by immunoblotting for TRIM5α and CPSF6, 25 d after nucleofection (Fig. 2b). Of note, the viability of the six-gene KO cells was around 20% lower compared to CD46 KO only or untreated WT cells (Fig. 2c). Importantly, while polyclonal resting six-gene KO CD4^+^ T cells remained negative for activation markers under standard IL-7 and IL-15-containing cultivation conditions (Fig. 2e), they could be readily activated two weeks after nucleofection (Fig. 2d,e) indicated by the cell surface expression of CD25/CD38/CD69 and HLA-DR upon stimulation with anti-CD3/CD28 monoclonal antibody-coated beads. Despite the simultaneous use of 12 gRNAs in the RNP complex, little (*CD46* and *CR1L*, Fig. 2f and Extended Data Table 1) or no off-targets effects were observed at the predicted two top coding off-target sites, despite extensive editing in the specific KO locus (Fig. 2f). Together, these results establish our technique as a versatile method for gene editing of resting human CD4^+^ T cells allowing multiplexing of KOs in this primary cell type without altering cells’ activation levels and preserving good viability for up to 6 weeks.

### The time to protein depletion in noncycling knockout CD4^+^ T cells is target specific

Next, we went on to conduct functional studies in polyclonal resting CD4^+^ T cells with various KOs. An important consideration in this context is that following efficient gene editing, functional alterations can only become apparent after the existing protein pool has been depleted. Owing to their individual turnover pathways, subcellular localization and resulting half-lives, the timespan required for a pronounced depletion is expected to be a specific property of each target protein and needs to be determined individually^19^. We compared the editing of three protein-encoding genes chosen as examples for different protein turnover rates and established their functions in viral infections or cell migration: CD46, described above, CXCR4, a seven-transmembrane chemokine cell surface receptor and HIV co-receptor^20^ and the HIV-1 restriction factor SAMHD1, a soluble deoxynucleoside triphosphate triphosphohydrolase localized in the nucleus and cytoplasm of resting CD4^+^ T cells^3^.

**Fig. 3:**
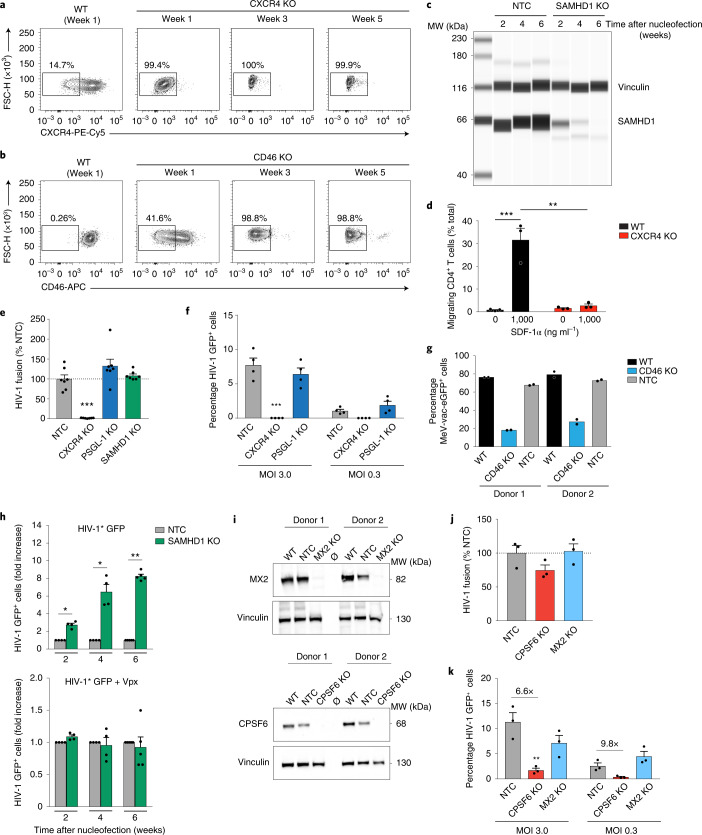
Phenotypic characterization of various single-gene KOs in polyclonal, resting CD4^+^ T cells. **a**,**b**, FACS density plots of surface-exposed CXCR4 (**a**) or CD46 (**b**) from one experiment at three time points (*n* = 3). **c**, WES immunoblot for SAMHD1 in cultivated KO or NTC cells. Vinculin was the loading control. One representative experiment is shown (*n* = 4). **d**, SDF-1α (CXCL12)-driven chemotaxis of cells 1 week after nucleofection. Mean ± s.e.m. are shown (*n* = 3). Statistics indicate significance by two-way ANOVA. *P* values were corrected for multiple comparison (Tukey) (***P = 0.0007; **P = 0.0010). **e**, The frequency of cells (NTC or indicated KOs) positive for cleaved CCF2 substrate after challenge with HIV-1 carrying BlaM-Vpr, indicative of HIV-1 fusion, was determined by flow cytometry. Mean ± s.e.m. are shown (*n* = 7). Statistics indicate significance by one-way ANOVA. *P* values were corrected for multiple comparisons (Dunnet) (***P = 0.0002). **f**, Cells with the indicated KOs were challenged 2 weeks after nucleofection with HIV-1 GFP at two MOIs and analyzed 3 d later. Mean ± s.e.m. are shown (*n* = 4). Statistics indicate significance by two-way ANOVA. *P* values were corrected for multiple comparisons (Tukey) (***P = ≤ 0.001). **g**, Cells were challenged 2 weeks after nucleofection with measles reporter virus MeV-vac-eGFP and analyzed for reporter expression by flow cytometry 1 d later. Means of technical duplicates are shown (*n* = 2). **h**, At the indicated weeks after nucleofection, SAMHD1 KO cells or NTC control cells were challenged with HIV-1* GFP either without Vpx (top) or carrying Vpx (+Vpx, bottom) and analyzed 3 d later by flow cytometry. Infection values for the NTC control were set to 1. Mean ± s.e.m. are shown (*n* = 5). Statistics indicate significance by two-tailed Mann–Whitney U-test. (2 weeks, *P = 0.0286; 4 weeks, *P = 0.0286; 6 weeks, **P = 0.0079). **i**, Immunoblots for MX2 (top) or CPSF6 (bottom) in cell lysates 4 weeks after nucleofection. Vinculin was the loading control. Two representative donors are shown (*n* = 4). Ø, empty lane. **j**,**k**, HIV-1 fusion (**j**) and HIV-1 GFP infection (**k**) in cells with the indicated KOs 4 weeks after nucleofection (as in **e** and **f**, respectively). Mean ± s.e.m. are shown (*n* = 3). Statistics indicate significance by two-way ANOVA. *P* values were corrected for multiple comparison (Dunnet) (**P = 0.0054). Source data

Following nucleofection of resting CD4^+^ T cells with gene-specific RNP complexes, CXCR4 surface expression became undetectable within 1 week of cultivation (Fig. 3a), whereas CD46 was only partially depleted at this time point and reached a near complete loss of surface exposure at week 2 (Fig. 1e) or 3 (Fig. 3b). The kinetic analysis of SAMHD1 levels by immunoblotting of gene-edited cells revealed an even slower decay of the lentiviral restriction factor with residual levels still detectable after 4 weeks and complete depletion of cellular SAMHD1 pools seen only 6 weeks after nucleofection (Fig. 3c and Extended Data Fig. 4b).

### Functional characterization of single-gene knockouts for cell migration and virus infection

**Extended Data Fig. 5:**
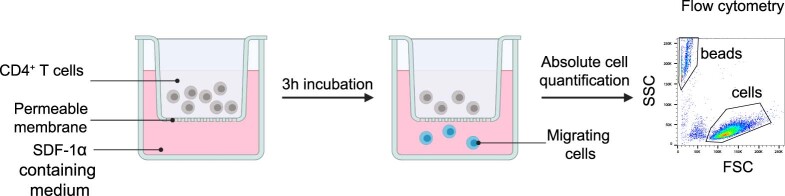
Schematic of T cell migration assay. Culture medium with or without supplementation with the natural CXCR4 ligand SDF-1-α was placed in the bottom chamber of a trans-well and CD4^+^ T cells (WT or CXCR4 KO) were added into the top chamber. After three hours, the absolute number of cells in the bottom chamber was quantified by flow cytometry using BD Trucount™ Tubes (schematic created with BioRender.com).

**Extended Data Fig. 6:**
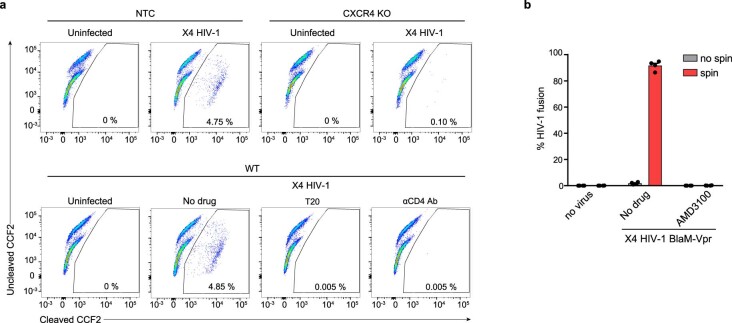
Principles of X4 HIV-1 fusion and infection assays in resting CD4^+^ T cells. Two different types of conditions for HIV-1 challenge were used depending on the scientific question, that is the target protein of interest and the step of the HIV replication cycle under investigation. **a**, First, to characterize the effect of KOs of either host dependency factors (CXCR4, CD4) or restriction factors (PSGL-1) for HIV binding and entry, we employed the well-established HIV fusion assay without spinoculation. Here, cells were exposed to the X4 HIV-1 BlaM-Vpr inoculum for 4 hours at 37 °C. Cells with either CXCR4 KO or nucleofected with NTC-gRNA were used. Representative dot plots of the flow cytometric detection of the CCF2 substrate cleavage by BlaM in viable cells after virion fusion are shown. As specificity controls, cells were pretreated with either the HIV-1 fusion inhibitor T20 or an anti-CD4 mAb. One representative experiment is shown (n = 7). **b**, Second, to characterize the role of potential cellular restriction factors (SAMHD1, MX2) or host dependency factors (CPSF6) at post-entry steps of the replication cycle (for example at reverse transcription, nuclear import, integration) we sought to efficiently overcome the natural restriction at virion entry and allow a high-level of virus delivery (see Fig. 3 and Fig. 5). In this context, we applied spinoculation at 650 g for 150 min at 37 °C. To prove that this is optimal for high-level virion delivery into resting CD4^+^ T cells, we performed a quantitative HIV-1 fusion assay. This spinoculation condition allowed X4 HIV-1 to enter into virtually every resting CD4^+^ T cell that expresses the co-receptor CXCR4, that is typically around 95% of the cell population. As specificity control, the small molecule inhibitor AMD3100, which blocks CXCR4-dependent HIV-1 fusion, was used. Means ± s.e.m. are shown (n = 4). Source data

This time-dependent loss of specific protein expression in resting CD4^+^ T cells had marked functional consequences. Depletion of CXCR4, which acts as receptor for the α-chemokine SDF-1α, abrogated the ability of CXCR4 KO cells to migrate across a Transwell membrane in response to its natural ligand (Fig. 3d and Extended Data Fig. 5). Similarly, CXCR4 KO cells no longer allowed fusion of replication-competent CXCR4-dependent (X4) HIV-1 particles carrying BlaM-Vpr chimeric proteins in a flow cytometry-based virion fusion assay^21,22^ (4.75% non-targeting control (NTC) cells versus 0.1% CXCR4 KO cells positive for cleaved CCF2 substrate, indicative of virion fusion) (Fig. 3e and Extended Data Fig. 6a). Consistently, CXCR4 KO cells were completely resistant to X4 HIV-1 green fluorescent protein (GFP) infection; <0.01% versus 7.7% of NTC cells expressed GFP reflecting viral gene expression from reverse-transcribed, integrated HIV-1 genomes 3 d after viral challenge (Fig. 3f) under conditions of highly efficient X4 HIV-1 fusion (Extended Data Fig. 6b).

**Extended Data Fig. 7:**
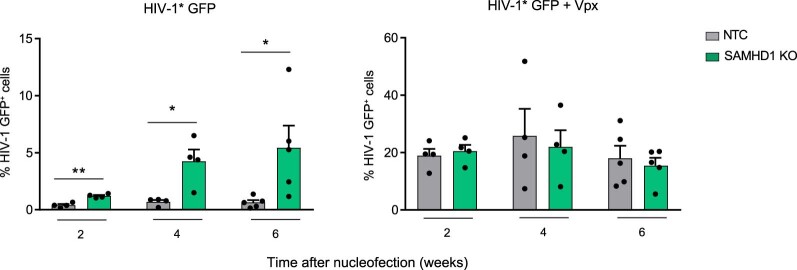
Time- and Vpx-dependent impact of SAMHD1 KO on HIV-1 infection in resting CD4^+^ T cells. SAMHD1 KO cells or NTC-nucleofected reference cells were challenged at the indicated weeks after nucleofection with either HIV-1* GFP without Vpx (left panel) or with Vpx (+ Vpx, right panel) and analyzed for GFP expression on day 3 by flow cytometry. Means ± s.e.m. are shown (n = 4-5). Statistics indicate significance by two-tailed paired *t*-test. (2 weeks, **P = 0.0035; 4 weeks, *P = 0.0294; 6 weeks*P = 0.0496). Source data

Further, consistent with the role of CD46 as the major receptor for measles morbillivirus (MeV)^18^, infection of resting CD4^+^ T cells carrying a CD46 KO with a MeV-GFP reporter virus was reduced up to 4.2-fold compared to untreated WT cells or NTC-nucleofected cells (Fig. 3g). Finally, to assess the impact of the progressive loss of the restriction factor SAMHD1 on HIV-1 infection in resting CD4^+^ T cells from healthy donors, we challenged SAMHD1 KO cells and NTC cells with a replication-competent X4 HIV-1 GFP reporter virus, which carries the Vpx-interaction motif in the Gag p6 protein (HIV-1* GFP), at different time points after nucleofection. Two weeks after nucleofection, reduced levels of SAMHD1 (Fig. 3c and Extended Data Fig. 4b) in SAMHD1 KO cells enhanced HIV-1* GFP infection significantly, by 2.5-fold, compared to NTC reference cells (Fig. 3h, top). This difference steadily increased for infections at subsequent time points reaching a maximum of ∼ninefold when cells were infected 6 weeks after nucleofection, at which time SAMHD1 was undetectable in KO cells (Fig. 3c,h (top) and Extended Data Fig. 4b). In contrast, HIV-1* GFP virions engineered to package the Vpx protein (HIV-1* GFP + Vpx) that recruits SAMHD1 to a cullin 4A-RING E3 ubiquitin ligase (CRL4-DCAF1), which targets the enzyme for proteasomal degradation^5,6^, did not show a significant difference of infection between SAMHD1 KO and NTC cells (Fig. 3h (bottom) and Extended Data Fig. 7 for absolute numbers of HIV-1 GFP^+^ cells). This is consistent with results from earlier work describing the effects of the lentivirus-encoded antagonist Vpx on SAMHD1 (refs. ^3,4,7^). Together, these results establish that RNP nucleofection and cultivation of resting CD4^+^ T cells according to our protocol enables the functional characterization of genetically depleted host cell factors for the infection of HIV-1 and other viruses (like MeV).

### CPSF6, but not PSGL-1 and MX2, is a critical factor for HIV-1 infection in resting CD4^+^ T cells

We next investigated the relevance of three host factors that have been shown to affect HIV-1 infection in other cell systems^23–26^, but whose role during infection of resting CD4^+^ T cells has not been defined. We generated KOs for either PSGL-1, MX2 or CPSF6. As described above, surface-exposed PSGL-1 was completely depleted 2 weeks after nucleofection. PSGL-1 has been shown to reduce virion infectivity and post-entry steps when expressed on HIV-1 producer or target cells (activated cells or cell lines), respectively^23,24,27^. For the first time, we were able to assess the potential impact of PSGL-1 on entry and post-entry steps in resting CD4^+^ T target cells. A CXCR4 KO was used as positive control, diminishing HIV-1 fusion and productive infection to background levels (Fig. 3e,f). In contrast, PSGL-1 KO cells retained their virion fusion capacity and permissivity to HIV-1 infection comparable to NTC control cells (Fig. 3e,f). Thus, PSGL-1 does not act as an entry or post-entry restriction factor for HIV-1 in resting CD4^+^ T target cells.

In KO cells, the host restriction factor MX2 and the host dependency factor CPSF6 became almost undetectable 4 weeks after nucleofection (Fig. 3i). Consistent with their reported role as cellular interactors of the HIV-1 capsid during nuclear entry of the pre-integration complex and proviral integration^25,26^, depletion of MX2 or CPSF6 proteins did not significantly impact on HIV-1 entry (Fig. 3j). To assess the effects of these KOs on productive HIV-1 infection, resting CD4^+^ T cells were challenged with HIV-1 GFP at two different multiplicities of infection (MOIs) and reporter gene expression was determined by flow cytometry 3 d later. Infection levels of MX2 KO and NTC cells were found to be largely comparable (Fig. 3k). While MX2 depletion increased HIV-1 infection rates in single-round analyses in other cell types between 3- and 11-fold^25,28^, our results suggest that MX2 is not a relevant HIV-1 restriction factor in resting CD4^+^ T cells. In contrast, loss of CPSF6 markedly reduced the efficacy of HIV-1 infection, by up to 9.8-fold, compared to the NTC reference in these primary cells (Fig. 3k). Previously reported inhibitory effects on HIV-1 infection upon depletion of the HIV-1 capsid-interacting CPSF6 in cell lines and primary macrophages were ∼threefold^26,29^, suggesting that the dependency of HIV-1 infection on CPSF6 is particularly high in resting CD4^+^ T cells. Together, these experiments validate RNP nucleofection of resting human CD4^+^ T cells as a rapid and effective method for gene editing, depletion of specific cellular factors and subsequent functional characterization in the context of HIV-1 infection in these thus far experimentally inaccessible primary human target cells.

### CRISPR-Cas9-mediated transgene knock in into multiple loci in resting CD4^+^ T cells

**Fig. 4:**
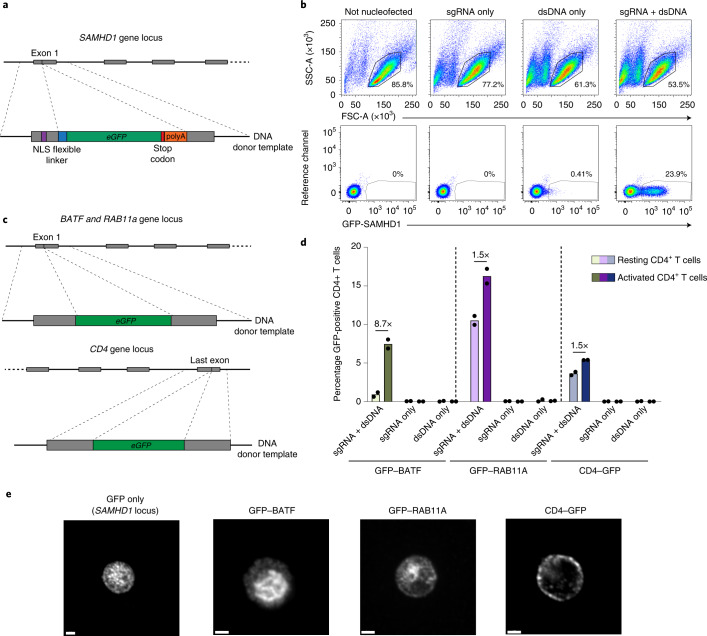
CRISPR-Cas9-mediated knock in of *eGFP* into different loci in resting CD4^+^ T cells. **a**, KI-targeting strategy to introduce *eGFP* into the *SAMHD1* locus. **b**, Cell viability and GFP expression after KI of the dsDNA cassette shown in **a**. Cells nucleofected with *s*gRNA2 only or with dsDNA only served as references. One representative experiment is shown (*n* = 3). **c**, KI-targeting strategy to introduce GFP to the N terminus of either BATF or RAB11A, or to the C terminus of CD4, in principle as reported for activated T cells^30^. **d**, GFP expression after KI of constructs from **c** analyzed by flow cytometry (*sgRNA* + dsDNA). Nucleofection of *sgRNA* only or dsDNA only served as references. CD4^+^ T cells were kept either resting or activated 3 d after nucleofection. Means of two independent donors are shown. **e**, Activated KI CD4^+^ T cells shown in **d** were fixed and stained with an antibody against GFP and analyzed by confocal microscopy. Representative micrographs from one experiment are shown (*n* = 3). Scale bars, 2 µm. Source data

**Extended Data Fig. 8:**
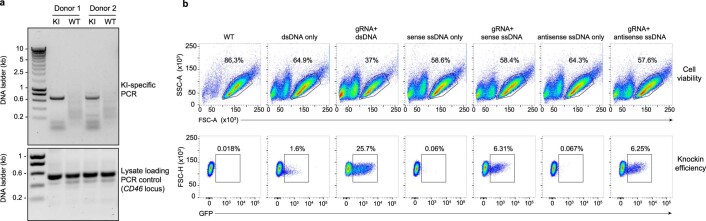
ssDNA as donor template improves cell viability, but reduces KI efficiency. **a**, KI CD4^+^ T cell cultures from Fig. 4b were lysed and a PCR specific for the *eGFP* integration into the *SAMHD1* locus was performed. Untreated WT cells served as reference. A PCR specific for the *CD46* locus was used as loading control (lower panel). **b**, GFP expression following KI (see also schematic in Fig. 4a). Density plots of flow cytometry analysis of viability (FSC/SSC, upper part) and GFP expression of resting CD4^+^ T cells two weeks after nucleofection. Cells were either left untreated (WT) or nucleofected with the indicated *SAMHD1*-gRNA2, dsDNA, ssDNA sense or ssDNA antisense templates alone or in combination. One µg of DNA template was used for each condition. One representative experiment is shown (n = 2).

Beyond the disruption of individual gene expression to identify their overall function, gene editing allows the introduction of specific mutations or protein tags for detailed mechanistic studies, for example by knock in (KI) via homology-directed repair (HDR)^30^. In activated T cells, KI levels of up to 69% have been reported^30^. To assess the suitability for locus-specific KIs in resting CD4^+^ T cells, we designed a strategy to modify the *SAMHD1* locus by insertion of an enhanced GFP-encoding reporter gene (*eGFP*) including a stop codon immediately downstream of the nuclear localization signal (NLS) of *SAMHD1*. In principle, this should result in GFP expression from the endogenous *SAMHD1* promoter, while transcription of *SAMHD1* itself is disrupted (Fig. 4a). As a donor template for HDR we used a double-stranded DNA (dsDNA) with around 550 bp homology arms from the region targeted by *SAMHD1*-gRNA2. While nucleofection of resting CD4^+^ T cells with dsDNA reduced cell viability in the absence of GFP reporter expression (Fig. 4b, top), nucleofection of dsDNA together with the RNP complex targeting the *exon 1* of *SAMHD1* resulted in GFP expression in >20% of these cells (Fig. 4b, bottom), indicating that the dsDNA donor template was successfully integrated and the GFP reporter was expressed. In parallel, the *eGFP* KI into *SAMHD1* was validated by a locus-specific PCR fragment amplification (Extended Data Fig. 8a). The use of single-stranded DNA (ssDNA) instead of dsDNA as donor template increased cell viability, in line with a recent report^30^, but markedly decreased KI efficiency (Extended Data Fig. 8b).

To examine whether this approach is widely applicable we performed KIs of the *eGFP* reporter cassette into the locus of three different genes (*BATF*, *RAB11A* and *CD4;* Fig. 4c) to generate fusion proteins, using reagents previously reported for activated T cells^30^. This protocol resulted in the expression of GFP fusion proteins from the endogenous promoters in resting CD4^+^ T cells 2 weeks after nucleofection as detected by flow cytometry (Fig. 4d) and by confocal microscopy in a typical subcellular localization (Fig. 4e; BATF, nuclear; RAB11A, intracellular compartment reminiscent of endosomes; CD4, plasma membrane). The KI efficiency and detectable expression of the GFP fusion proteins in resting cells varied from 0.9% (BATF) to 10.5% (RAB11A) (Fig. 4d), most likely reflecting differences in locus-specific HDR efficiency related to the sequence of the homology arms, gRNA efficiency or the endogenous promoter activity. Of note, the expression of GFP-BATF was induced upon T-cell activation (8.7-fold, Fig. 4d), in line with a previous report^31^, providing an example for an endogenous fluorescent reporter of activation in primary human T cells.

### Knock in of *eGFP* upstream of *SAMHD1* allows studies into the physiological interplay of the cellular restriction factor, HIV-1 and an accessory lentiviral protein

**Fig. 5:**
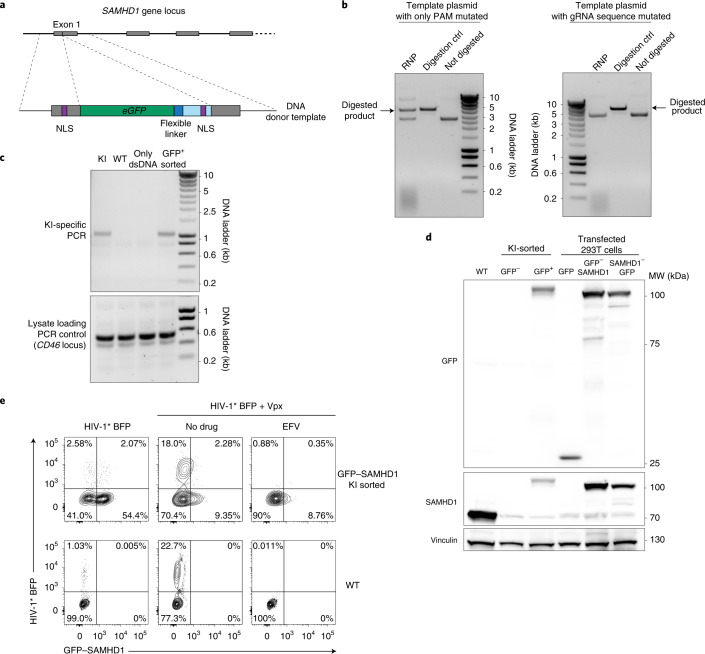
GFP-SAMHD1 endogenously expressed in resting CD4^+^ T cells is functional in the context of HIV-1 infection and degraded by particle-packaged Vpx. **a**, KI-targeting strategy to introduce a N-terminal GFP fusion into the endogenous *SAMHD1* locus. The dsDNA template from **a** was introduced into a plasmid and digested using the *SAMHD1*-gRNA2-containing RNP. **b**, Digestion of the dsDNA template with either PAM sequence mutated only (left) or the sequence complementary to *SAMHD1*-gRNA2 mutated completely, including the PAM sequence (right). No digestion or *BstB*I digestion (digestion ctrl) served as references. One experiment is shown (*n* = 2). **c**, GFP-positive KI resting CD4^+^ T cells were sorted by flow cytometry and lysed and a PCR specific for the *eGFP* integration into the *SAMHD1* locus was performed. Untreated WT cells and cells nucleofected with dsDNA template only served as references. A PCR specific for the *CD46* locus was used as loading control (bottom). One experiment is shown (*n* = 2). **d**, Sorted cells from **c**, either positive or negative for GFP, were immunoblotted for both SAMHD1 and GFP. WT cells served as reference; vinculin was the loading control. The 293T cells transfected with expression plasmids encoding either GFP, GFP–SAMHD1 or SAMHD1–GFP served as references. One representative experiment is shown (*n* = 2). **e**, Cells were challenged with equivalent infectious units of HIV-1* BFP virions with (+Vpx) or without the lentiviral SAMHD1 antagonist Vpx and analyzed by flow cytometry on day 3. Reverse transcriptase inhibitor EFV served as a specificity control. One representative experiment is shown (*n* = 2).

**Extended Data Fig. 9:**
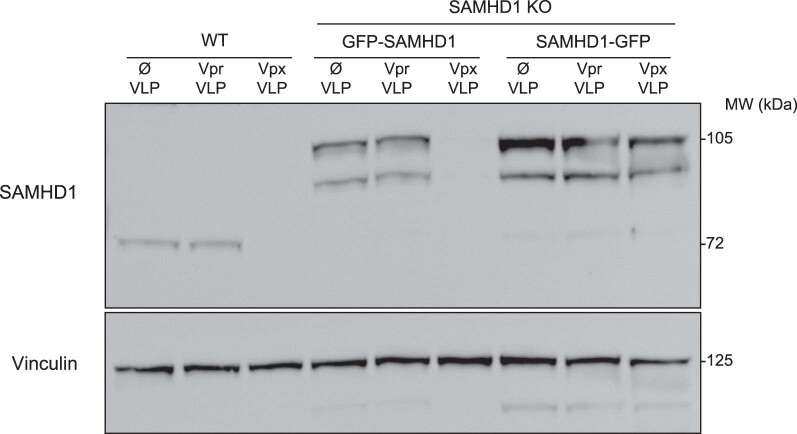
The GFP-SAMHD1 fusion protein, expressed from the SAMHD1 locus, can be degraded by the lentiviral Vpx protein. Monoclonal SAMHD1 KO cells were generated in human glioblastoma cell line LN18. Next, SAMHD1 KO LN18 cells were stably transfected with linearized plasmids encoding for either GFP-SAMHD1 or SAMHD1-GFP. GFP-positive cells were sorted by flow cytometry 4 weeks after transfection. To deliver Vpx into cells, WT LN18 and SAMHD1 KO cells expressing either GFP-SAMHD1 or SAMHD1-GFP were challenged with VSV- G pseudotyped virus like particles (VLPs) with incorporated lentiviral Vpx (Vpx-VLPs). Empty VLPs (Ø) or VLPs with incorporated Vpr (Vpr-VLPs) were used as negative controls. After VLP treatment, cells were collected and SAMHD1 immunoblots performed. Vinculin served as loading control. Endogenous SAMHD1 in WT cells and stably expressed GFP-SAMHD1 were degraded by Vpx. In contrast, SAMHD1-GFP was protected from Vpx degradation likely due to steric hindrance. Experiment performed only one time.

**Extended Data Fig. 10:**
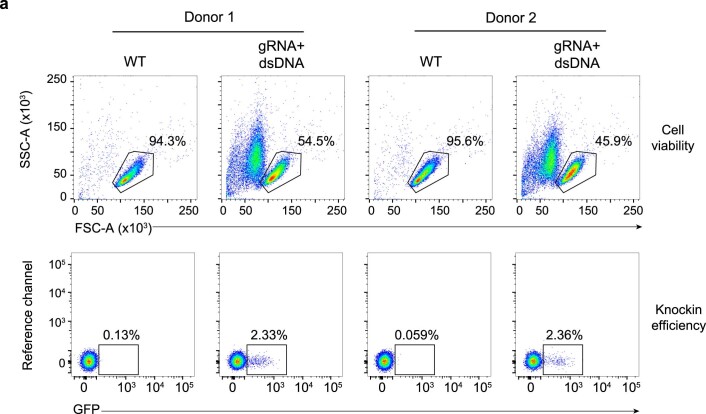
Efficiency of the GFP-SAMHD1 KI approach in resting CD4^+^ T cells. Density plots of flow cytometric analyses of viability (FSC/SSC, upper panels) and GFP expression (lower panels) of nucleofected cells two weeks after nucleofection and prior to sorting (see also Fig. 5e). WT cells served as reference. Data from two independent donors are shown.

To apply the KI approach to a functional analysis in the context of HIV-1 infection in resting CD4^+^ T cells, we introduced the *eGFP* gene upstream of the *SAMHD1* locus to generate a GFP-SAMHD1 fusion protein (Fig. 5a), which is a Vpx-degradable (Extended Data Fig. 9) and enzymatically active analog of non-tagged SAMHD1. Of note, the new dsDNA template carries the *SAMHD1*-gRNA2 binding site (Fig. 5a). To avoid cleavage of the donor template by the RNP, the PAM sequence in the dsDNA was mutated and a corresponding plasmid tested for gRNA in vitro digestion. Unexpectedly, mutating the PAM sequence alone decreases the cutting efficiency, but this was not sufficient to fully abrogate in vitro cleavage of the DNA (Fig. 5b, left), in contrast to a recent report^32^. This problem was overcome by mutating the complete gRNA-binding sequence of *SAMHD1*-gRNA2, while preserving the amino acid sequence (Fig. 5b, right). Five days after nucleofection this KI strategy resulted in 2.3% viable, GFP-positive cells (Extended Data Fig. 10), which were subsequently enriched by cell sorting. The sorted cell population carried the KI cassette in the *SAMHD1* locus as verified by a locus-specific PCR amplification specific for the *eGFP* integration (Fig. 5c). Similarly, immunoblots of the sorted, GFP-positive cell population showed reactivity of the GFP–SAMHD1 fusion protein (∼100 kDa) to both anti-GFP and anti-SAMHD1 antibodies (Fig. 5d). Sorted, GFP-negative cells and plasmid-transfected 293T cells served as references.

For functional analysis, sorted GFP–SAMHD1-expressing resting CD4^+^ T cells or unmanipulated, sorted WT reference cells were challenged with X4 HIV-1* BFP virions with Vpx (+Vpx) or without and analyzed on day 3 after virus challenge by flow cytometry. As expected^3,4^, both cultures were largely refractory to infection with X4 HIV-1* BFP (Fig. 5e, top quadrants, left, 2.58% and 0.88%) and expression of GFP–SAMHD1 remained intact compared to WT cells (Fig. 5e, left). In contrast, infection with HIV-1* BFP virions containing Vpx was efficient, yielding 20.28% of BFP^+^CD4^**+**^ T cells, the majority of which also showed a strong depletion of GFP–SAMHD1 (Fig. 5e, top quadrants, middle top). Consistent with the model that virion-incorporated Vpx rapidly targets endogenous SAMHD1 for degradation, depletion of GFP–SAMHD1 was readily observed, yet addition of the reverse transcriptase inhibitor efavirenz (EFV) still prevented progression of the replication cycle and productive infection (Fig. 5e, right). Thus, this KI strategy enables the introduction of a functional reporter system into an endogenous locus to study virus-host interactions at single-cell resolution in quiescent T cells.

## Discussion

Together, our results establish a set of protocols that overcomes the resistance of resting human CD4^+^ T cells to genetic manipulation and combined with improved cultivation conditions enables mechanistic studies of this primary cell type. These protocols allow a high KO efficiency with the option of simultaneous multi-gene KOs, enhanced cell viability while preserving a resting state of T cells and the KI of fluorescently modified genes into various endogenous loci to conduct functional characterizations of interest. The RNP nucleofection protocol, which combines two to three pre-tested gRNAs per target gene, in combination with optimized cell culture conditions, resulted in remarkably high editing rates (typically >98%). This approach yielded cell populations, in which protein expression of the specific targets was reduced to undetectable levels. Further characterizations revealed that this reduction in protein expression also translated into a loss of function, demonstrating that this KO protocol facilitates insight into physiological virus–host interactions. Its efficacy and speed alleviate the need for any selection process and allows the analysis of a polyclonal, potentially heterogeneous primary cell population.

Notably, our protocol did not result in CD4^+^ T-cell activation as assessed by activation marker expression and various markers of cell proliferation and gene-edited cells maintained their general resistance to HIV-1 infection as well as their sensitivity to the lentiviral Vpx protein for infection enhancement. We found that cultivation of resting CD4^+^ T cells in the presence of low concentrations of human IL-7 and IL-15 preserved cell viability for up to 6 weeks without inducing cell proliferation or expression of activation markers. This provided the basis for efficient gene editing and functional downstream analyses following loss of the respective target proteins of interest. A recent review^33^, discussed the importance of IL-7 and IL-15 for the viability of primary mouse T cells ex vivo. Notably, this cytokine combination induces both T-cell activation and proliferation, referred to as T-cell receptor-independent homeostatic proliferation, in mice, suggesting a species-specific response to these ILs.

In contrast to the commonly used post-activation protocols, cells edited by this protocol maintain the key physiological properties of resting CD4^+^ T cells. Our approach allowed the simultaneous depletion of up to six individual proteins or the KI of a functional reporter gene. The workflow described herein thus overcomes major limitations of a previously reported protocol for gene editing of resting CD4^+^ T cells^12^ in that it (1) maximizes KO efficiency at enhanced cell viability, (2) preserves cells’ resting activation state over weeks of culture and (3) enables simultaneous multi-gene KOs as well as KIs of modified genes. This approach opens avenues not only for the functional characterization of individual gene products in bulk populations of truly resting CD4^+^ T cells, but also for more complex mechanistic studies by targeting entire cellular pathways, analysis of functional redundancy and compensatory mechanisms as well as introduction of specific mutations, tags and functional reporters.

These protocols enable robust gene editing combined with functional characterization of resting CD4^+^ T cells. This provides methodology to decipher the role of specific restriction factors, host dependency factors and nucleic acid sensors for determining the balance of resistance and susceptibility of these cells to HIV-1 infection, the establishment of viral latency and why the infection in resting cells is not productive^34–36^.

Some limitations toward high-throughput applications remain. First, the specific time point at which the protein of interest is sufficiently depleted for functional analysis needs to be determined for each target gene. Second, and owing to the nonproliferative nature of resting CD4^+^ T cells, the number of cells per donor that is available for analysis remains somewhat limited. In summary, applying these KO and KI protocols will yield insights into the processes governing infection, latency, reactivation and immune recognition of HIV-1, but also other T-cell tropic viruses such as measles virus, human T-lymphotropic virus, human herpesviruses 6 and 7, human cytomegalovirus and human herpes simplex virus type 2 (ref. ^37^), as well as virus-unrelated studies of activation, proliferation and differentiation of this cardinal human cell type.

## Methods

### Primary human CD4^+^ T cells

CD4^+^ T cells were isolated from heparinized blood retained in leukocyte reduction system chambers from healthy blood donors with approval by the Ethics Committee of the LMU München (project no. 17-202 UE), in principle as reported^38^. In brief, blood cells were diluted with PBS (Gibco) and CD4^+^ T cells were isolated via EasySep Rosette Human CD4^+^ T cell enrichment kits (STEMCELL Technologies) according to the manufacturer’s protocols. Resting CD4^+^ T cells were kept in RPMI 1640 GlutaMAX (Gibco) supplemented with 10% (v/v) fetal bovine serum (FBS; Sigma) and penicillin/streptomycin (100 IU ml^−1^; Thermo Fisher Scientific) with the addition of IL-7 and IL-15 (2 ng ml^−1^ each; Peprotech) and cultivated in 96-well flat-bottom plates at a cell density of 1 × 10^6^ cells ml^−1^. The culture medium was replaced every 3 d. For activation, Dynabeads Human T-Activator CD3/CD28 (Gibco) were added to CD4^+^ T cells at a ratio of 1:10 (bead to T cell) and kept in medium containing IL-2 (50 IU ml^−1^; Biomol). Fresh beads were added to the culture every other week.

### Cell lines

Human T-cell line SUP-T1 (DSMZ, ACC140) was cultivated in RPMI 1640 GlutaMAX (Gibco) supplemented with 10% (v/v) FBS and penicillin/streptomycin (100 IU ml^−1^). The 293T cells (DSMZ; ACC 635) and LN18 were cultivated using DMEM GlutaMAX (Gibco) containing the same additives.

### Knockout generation in resting human CD4^+^ T cells

Freshly isolated CD4^+^ T cells (2 × 10^6^) were washed twice with PBS and resuspended in 20 µl buffer P3 (Lonza; V4XP-3032). In parallel, synthetic sgRNAs (Synthego) were incubated together with recombinant NLS-Cas9 (IDT; 1081059) for 10 min at room temperature, at a ratio of 1:2.5 (40 pmol Cas9 protein per 100 pmol gRNA) to form the CRISPR–CAS9–gRNA RNP complex. The CAS9–gRNA mix was diluted with sterile filtered (0.22 µm) PBS to reach a final concentration of 20 µM RNPs. For single gRNA editing, 5 µl of the 20 µM RNPs were then mixed with the cell suspension and transferred into a 16-well reaction cuvette of the 4D-Nucleofector System (Lonza). For efficient KO of individual targets, a mix of two specific, pre-validated gRNAs was used, if not indicated differently. Here, only 2 µl of RNP complexes for each gRNAs were used. For co-editing of up to six genes, only 0.5 µl of each RNP complex were used. Cells were nucleofected using program EH-100 on the 4D-Nucleofector system^12^. Then, 100 µl of pre-warmed RPMI (without supplements) was added to each well and cells were transferred to 48-well plates and allowed to recover for 10 min at 37 °C. Subsequently, complete culture medium supplemented with IL-7 (Peprotech; 200-07) and IL-15 (Peprotech; 200-15) (2 ng ml^−1^ each) was added. A list of gRNA sequences used in this study is provided in Extended Data Table 1.

### TIDE analysis and Illumina MiSeq

One week after nucleofection, cells (5 × 10^4^) were collected and lysed in lysis buffer (20 µl) (1 mM CaCl_2_, 3 mM MgCl_2_, 1 mM EDTA, 1% Triton X 100 and 10 mM Tris, pH 7.5) with the addition of proteinase K (20 µg ml^−1^; Thermo Fisher Scientific). This cell lysate was incubated at 65 °C for 20 min, followed by 95 °C for 15 min and then stored at −20 °C until the PCR specific for the CRISPR/Cas9 target sites was performed. Then, 1 µl of cell lysate was used as a PCR template. For Tracking of Indels by DEcomposition (TIDE) analysis, the PCR reaction contained 5 µl 5× high-fidelity PCR buffer (Thermo Fisher Scientific), 0.5 µl dNTPs (10 mM stock; Thermo Fisher Scientific), 1.2 µl forward primer (10 µM stock), 1.2 µl reverse primer (10 µM stock), 0.25 µl Phusion (NEB), 1 µl lysate and 15.85 µl H_2_O. The PCR cycler (Thermo Fisher Scientific) settings were 95 °C for 5 min, followed by 35 cycles at 95 °C for 20 s, 58–65 °C (depending on the specific primer pair) for 30 s and 72 °C for 40 s, with the final step at 72 °C for 3 min. The PCR was then cleaned up using the NucleoSpin Gel and PCR clean-up columns (Macherey-Nagel), followed by Sanger sequencing that was performed by Eurofins. Sequencing results were analyzed with the TIDE webtool (http://tide.nki.nl/)^39^. For Miseq, 1 µl of lysate was used to perform PCR-I and subsequently PCR-II followed by Illumina MiSeq analysis as described previously^40^. The results of the MiSeq were analyzed with the Outknocker 2.0 webtool (http://www.outknocker.org/outknocker2.htm).

For the analysis of off-target sites, cells were collected 1 week after nucleofection and lysed. The top two off-target sites in open reading frames were selected using the Synthego CRISPR design tool (https://design.synthego.com). A PCR specific for each off-target site was performed and analyzed by Illumina Miseq. Primers used are listed in Extended Data Table 1.

### Immunoblotting

CD4^+^ T cells were collected and washed twice with PBS. Cell pellets were resuspended in RIPA buffer supplemented with proteinase inhibitors (Roche) and phosphatase inhibitors (Thermo Fisher Scientific) and kept on ice for 30 min followed by freezing at −80 °C. Cell lysates were thawed on the day of the experiment, spun clear at 10,000*g* for 10 min at 4 °C and lysates were transferred to new tubes. The protein concentration was quantified by BCA assay (Thermo Fisher Scientific), following the manufacturer’s protocol. Lysate samples were separated by tris-glycine denaturing SDS–PAGE (Thermo Fisher Scientific). Proteins were blotted onto 0.2-mm nitrocellulose membranes (GE Healthcare), blocked in 5% milk (Roth) in TBS-T for 1 h and incubated with the indicated primary antibody in 1% BSA/TBS-T or 5% milk, depending on the antibody used and subsequently with the corresponding secondary antibodies for 1 h (1:10,000 dilution in 5% milk). ECL (Bio-Rad) was used as substrate and the chemiluminescent signals were detected on a Fusion Fx (Vilber). The following human-specific antibodies were used: anti-SAMHD1 (proprietary chicken monoclonal antibody of the Keppler laboratory), anti-CPSF6 (rabbit, polyclonal, Cell Signaling, cat. no. 75168S) 1:1,000 dilution, anti-vinculin (mouse, hVIN-1, Sigma Aldrich, cat. no. V9264) 1:2,000 dilution, anti-MX2 (rabbit, polyclonal, Novus Biologicals, cat. no. NBP1-81018) 1:250 dilution, anti-TRIM5α (rabbit, clone D6Z8L, Cell Signaling, cat. no. 14326S) 1:1,000 dilution, anti-SAMHD1 (mouse, clone OTI3F5, Origene, cat. no. TA502024) 1:250 dilution and anti-GFP (rabbit polyclonal, Chromoteck, PABG1-20) 1:1,000 dilution. The following HRP-conjugated secondary antibodies were used in a dilution of 1:10,000: goat anti-mouse IgG (rat adsorbed, polyclonal, Bio-Rad, cat. no. STAR77), goat anti-chicken IgY (H&L, polyclonal, Abcam, cat. no. ab6877) and goat-IgG anti-Rabbit IgG (H + L, polyclonal, Dianova, cat. no. AFK-600). In six-gene KO cells (Fig. 2b) TRIM5α and CPSF6 expression was analyzed by consecutive re-probing of nitrocellulose membranes, followed by staining for vinculin (loading control). For immunoblots of GFP–SAMHD1 KI CD4^+^ T cells, due to the limited number of cells available, cells were activated first with Dynabeads Human T-Activator CD3/CD28 and IL-2 for 1 week to allow expansion, subsequently collected and sorted with the FACSAria Fusion cell sorter (BD) and lysed as described above. Gating strategies are described in Extended Data Fig. 1d and Supplementary Data. Full-length blots are provided as Source Data.

### WES system western blot

Cells were lysed and the protein concentration was quantified as described above. Then, 0.6 µg of total protein was evaluated by separation and immunodetection employing the WES system (ProteinSimple) with a separation matrix of 12–230 kDa. The primary antibodies used for the WES evaluation detect SAMHD1 (proprietary mouse monoclonal antibody of the Keppler laboratory, clone H154, produced by Eurogentec; 1:200 dilution) and vinculin (mouse, hVIN-1, Sigma Aldrich, cat. no. V9264; 1:2,000 dilution). Full-length blots are provided as Source Data.

### Antibodies, cell staining and flow cytometry

Cells were collected, washed once with PBS and resuspended in 50 µl staining solution (FACS buffer (PBS, 1% FBS and 2 mM EDTA) containing specific antibodies) and kept for 20 min at 4 °C. Then, cells were washed and resuspended in 100 µl FACS buffer. The following antibodies were used: anti-CD46 (BV421; BD, cat. no. 743776; PE; BD, cat. no. 564252, clone E4.3; APC; BD, cat. no. 352405, clone TRA-2-10), anti-CXCR4 (BV421, BD, cat. no. 562448; PE-Cy.5, BD, cat. no. 555975, clone 12G5; PE-Cy7, BioLegend, cat. no. 306514, clone 12G5), anti-PSGL-1 (Alexa Fluor 647, BioLegend, cat. no. 328809, clone KPL-1; BV421, BD, cat. no. 743478, clone KPL-1), anti-CD25 (BV421, BD, cat. no. 562442; APC, BD, cat. no. 555434, clone M-A251), anti-CD69 (BV421, BD, cat. no. 562884; APC, BD, cat. no. 555533, clone FN50), anti-CD4 (PE-Cy7, BioLegend, cat. no. 300512´, clone RPA-T4; APC, BD, cat. no. 555349, clone RPA-T4), anti-CD38 (PE, BD, cat. no. 555460, clone HIT2), anti-HLA-DR (FITC, BD, cat. no. 347400, clone L243), goat anti-mouse IgG (H + L) (Alexa Fluor 647, Invitrogen, cat. no. A-21236), polyclonal rabbit anti-GFP (Chromoteck, PABG1-20), polyclonal goat anti-rabbit IgG (H + L) (Alexa Fluor 647, Invitrogen, cat. no. A-21245). The following dyes were used: LIVE/DEAD Fixable Near-IR Dead Cell Stain kit (Invitrogen), Click-iT EdU Alexa Fluor 647 kit (Thermo Fisher Scientific) and CellTrace CFSE Cell Proliferation kit (Thermo Fisher Scientific) following the manufacturer’s protocol. For Pyronin Y (Sigma Aldrich) staining, cells were washed once with PBS and resuspended in 70% ethanol and stored at −20 °C for at least 2 h. After this time, cells were washed with FACS buffer and resuspended in staining solution (500 µl FACS buffer, 2 µg Pyronin Y and 1 µg Hoechst 33342) for 20 min at room temperature. Hoechst 33342 was used to prevent Pyronin Y binding to DNA, reducing the background^41^. Finally, cells were washed twice and resuspended in 80 µl FACS buffer. Stained cell suspensions were analyzed with the BD FACS Lyric (BD) using FlowJo software (BD). In general, forward and side scattering of light (FSC/SSC) were used to identify live cells by flow cytometry. For the analysis shown in Fig. 1b, a cell aliquot was analyzed once a week by staining with LIVE/DEAD Fixable Near-IR Dead Cell Stain to assess culture viability.

### HIV-1 plasmids

For HIV infection, the HIV-1 GFP proviral clone NLENG1-IRES^42^ was used, referred to as HIV-1 GFP in the current study. The Vpx-binding motif DPAVDLL from SIVmac Gag was introduced into the p6 of NLENG1-IRES (referred to as HIV-1* GFP in this study; GenBank accession no. OK558601), obtained from the plasmid HIV-1* NL4-3 (ref. ^3^) using the restriction sites *Sph*I and *Age*I. For packaging of Vpx into virions, the Vpx expression construct pcDNA3.1 Vpx SIVmac239-Myc was used and the pcDNA3.1 empty vector served as negative control^3^. GFP was replaced by mtagBFP to obtain HIV-1* BFP (GenBank accession no. OK558602). An insert obtained from pCDH-mtagBFP vector^43^ was introduced into the HIV-1* GFP using the restriction sites *Sph*I and *Age*I. For the virion fusion assay, the R5 HIV-1 proviral clone HIVivo^44^, kindly provided by M. Nussenzweig (Laboratory of Molecular Immunology, The Rockefeller University, New York, NY, USA), was used in combination with pCMV-BlaM-Vpr during virus production (see below). X4 HIVivo (GenBank accession no. OK589863) was generated introducing the X4 envelope gene from NLENG1-IRES into the R5 HIVivo backbone using the restriction sites *EcoR*I and *Hpa*I. Snapgene was used to design the cloning strategy and the primers needed.

### HIV-1 production

Sucrose-cushion-purified HIV-1 stocks were produced as previously described^45^. HIV-1* GFP virus stocks, carrying virion-packaged Vpx, were produced by co-transfection of the proviral HIV-1* GFP DNA and the indicated Vpx expression constructs^7^. In brief, 293 T cells were seeded at a density of 8 × 10^6^ cells in a 15-cm dish. After 24 h, cells were co-transfected with a mixture of 37.5 µg HIV-1 plasmid and 112.5 µl of L-PEI (3 µl of L-PEI for every µg of DNA, stock concentration of 1 µg µl^−1^; Polysciences) in DMEM without any additives for 30 min. After this time, the DNA/PEI solution was added to the cells. After 72 h, the supernatant was collected and virus was purified via sucrose-cushion centrifugation. For virions to incorporate Vpx, the transfection was performed as described above, but using 37.5 µg of HIV-1* GFP or HIV-1* BFP together with 18.75 µg of pcDNA-Vpx (SIVmac239-Myc) or the corresponding pcDNA3.1 empty control vector. For virus production for the virion fusion assay, the transfection was performed as described above, combining 37.5 µg of X4 HIVivo and 12.5 µg of pCMV-BlaM-Vpr.

### HIV-1 fusion assay

T cells were incubated with virions containing BlaM-Vpr at 37 °C for 4 h. Subsequently, cells were washed twice in CO_2_-independent medium (Thermo Fisher Scientific) and then loaded with CCF2/AM dye (Thermo Fisher Scientific), as described previously^21,22^. Briefly, 2 µl of CCF2/AM (1 mM) was mixed with 8 µl of solution B and 10 µl of probenecid (250 mM stock; MP Biomedicals) in 1 ml of CO_2_-independent medium supplemented with 10% FBS (v/v). Cells were incubated in 100 µl of loading solution for 16 h at room temperature. Cells were then washed twice with PBS and fixed with 4% (v/v) paraformaldehyde (PFA) for 1.5 h. Subsequently, cells were washed and resuspended in FACS buffer. The shift in emission fluorescence of CCF2 after cleavage was monitored by flow cytometry.

### HIV-1 infection

The titer of individual virus stocks was determined on SUP-T1 cells using virus-encoded GFP or BFP signals measured by flow cytometry as readout for productive infection. Primary resting CD4^+^ T cells were infected with virus stocks at different MOIs as indicated for each experiment. Where indicated, infections were performed either by co-incubation of virus and cells without additional centrifugation or by spinoculation for 2.5 h at 650*g* and 37 °C. After 3 d, cells were washed twice with PBS and fixed with 4% (v/v) PFA for 1.5 h. Cells were then washed and resuspended in FACS buffer. The percentage of GFP- or BFP-positive cells was monitored by flow cytometry. Drug or antibody controls were added to cells 30 min before HIV-1 challenge. The following drugs were used: EFV (stock, 10 mM; Sigma Aldrich), AMD3100 (stock, 16 µg ml^−1^; Sigma Aldrich), anti-CD4 clone SK3 (stock, 25 µg ml^−1^; (cat. no. 344602) BioLegend) and T20 (stock, 90 mg ml^−1^; Enfuvirtid; Roche). For infection of GFP–SAMHD1 KI CD4^+^ T cells, GFP-positive resting CD4^+^ T cells, 1 week after nucleofection, were sorted using a FACSAria Fusion cell sorter (BD), allowed to rest for 16 h and then challenged with virus.

### Chemokine-migration assay

Resting CD4^+^ T cells were used 1 week after nucleofection for this assay (CXCR4 KO or NTC). After removing the membrane from the Transwell system, 500 µl of RPMI medium supplemented with 0.2 % of FCS and the chemokine SDF-1α (1,000 ng ml^−1^; Peprotech) were added at the bottom of the 6.5-mm Transwell with 3.0-µm pore (24-well plate; Corning). The polycarbonate membrane was added into the corresponding wells and for each condition, 200 µl containing 2.5 × 10^5^ cells were transferred to the top of the membrane. The 24-well plate was incubated at 37 °C for 3 h. Subsequently, the membrane was removed and the total number of cells in the bottom chamber of the Transwell was quantified by flow cytometry using BD Trucount Absolute Counting Tubes.

### Measles virus infection

CD46 KO and WT reference resting CD4^+^ T cells were challenged with a measles GFP reporter virus (MeV-vac-eGFP), Schwarz-ATU-GFP^46^, at an MOI of 1. After 24 h, cells were stained with an APC-conjugated antibody to detect CD46 surface expression (clone TRA-2-10; BioLegend) and analyzed by flow cytometry. The MeV-vac-eGFP stock was generated as described^46^.

### Production of knock in DNA templates

The plasmids containing the donor DNA template for each KI approach were synthesized by Twist Bioscience (pTwist KI-Template GFP; GenBank accession no. OK558599) and pTwist KI-Template GFP-SAMHD1 (GenBank accession no. OK558600). The DNA template was amplified by PCR from these plasmids using specific primers. The PCR reaction contained 5 µl 5× High-fidelity PCR buffer (Thermo Fisher Scientific), 5 µl 5× GC PCR buffer (Thermo Fisher Scientific), 1 µl dNTPs (10 mM stock; Thermo Fisher Scientific), 1.5 µl dimethylsulfoxide, 2.5 µl forward primer (10 µM stock), 2.5 µl reverse primer (10 µM stock), 1 µl Phusion (NEB), 1 µl (10 ng) plasmid and 30.5 µl H_2_O. The primers used for the different KI templates are reported in Extended Data Table 1. The PCR cycle settings were 95 °C for 5 min, followed by 35 cycles at 95 °C for 30 s, 58 °C for 30 s and 72 °C for 90 s, with the final step at 72 °C for 5 min. For ssDNA production, a PCR product containing a phosphate group on one of the two strands is required (see below). For this reason, additionally, either the forward primer or the reverse primer were replaced with a primer of the same sequence but with an additional phosphorylation. The ssDNA was produced with the Guide-it Long ssDNA Production System (Takara Bio) according to the manufacturer’s protocol. After the PCR, a PCR clean-up was performed with the NucleoSpin Gel and PCR clean-up (Macherey-Nagel) according to the manufacturer’s protocol. Finally, the DNA concentration was determined by NanoDrop (Thermo Fisher Scientific). Sequences of KI templates used are listed in Extended Data Table 1. To knock in *eGFP* into the *SAMHD1* locus (Fig. 4a), a dsDNA template with homology arms of around 550 bp each, including an *eGFP* reporter gene followed by a stop codon and a polyadenylation (polyA) signal was used to disrupt endogenous SAMHD1 expression.

### In vitro digestion of knock in DNA templates

To test whether the KI dsDNA templates are cleaved by the gRNAs used to generate the double-strand break in the target cell genome, an in vitro restriction was performed. The Cas9–RNP complex (1 µM) as well as a single-cutter restriction enzyme (*BstB*I) for the specific plasmid were used and samples were incubated at 37 °C for 2 h. Subsequently, 1 µl of proteinase K (20 mg ml^−1^) was added and samples were incubated at 56 °C for 10 min, separated and visualized on an agarose gel (1 %).

### Knock in of resting CD4^+^ T cells

For KIs, the same nucleofection conditions as for the KO generation were used (P3 buffer and program EH-100; Lonza). In addition to the RNP, the donor DNA template was added to the P3 cell suspension at 1 µg, unless stated otherwise. Additional information on the overall strategies to generate KIs into different loci in resting CD4^+^ T cells, including DNA templates, gRNAs and primers is included in Extended Data Table 1.

### Confocal microscopy

Resting CD4^+^ T cells were used 2 weeks after nucleofection (KO for CD46, CD4, PSGL-1 or CXCR4). Cells were stained with antibodies against either CD46 (APC clone, TRA-2-10; BioLegend), CXCR4 (APC clone, 12G5; BD), PSGL-1 (Alexa Fluor 647, KPL-1; BD) or CD4 (APC clone, RPA-T4; BD). Cells were collected, washed once with PBS and resuspended in 50 µl staining solution (FACS buffer and specific antibodies) and kept for 20 min at 4 °C. To amplify the signal for CD46 a secondary antibody was used (goat anti-mouse IgG (H + L), Alexa Fluor 647; (cat. no. A-21236), Invitrogen). After this time, cells were washed, fixed with 4% PFA/PBS for 10 min at room temperature and washed again. Cell were then mounted with ProLong Diamond Antifade Mountant (Thermo Fisher Scientific) and analyzed with a spinning disk confocal microscope (Nikon). For the KI experiments, activated KI cells were used 2 weeks after nucleofection. Cells were washed, as described above and fixed with BD Cytofix for 10 min at room temperature. After washing, cells were permeabilized with Perm Buffer III (BD) for 10 min on ice. Cells were then washed twice with Perm/Wash buffer (BD) and resuspended in Perm/Wash buffer containing the primary antibody (GFP clone, PABG1; Chromoteck) for 30 min on ice. Cells were then washed twice with Perm/Wash buffer and resuspended in Perm/Wash buffer containing the secondary antibody (anti-rabbit IgG (H + L) and Alexa Fluor 647 (Invitrogen, cat. no. A-21236)) for 30 min on ice. Cells were then washed twice and mounted with ProLong Diamond Antifade Mountant and analyzed by spinning-disk confocal microscopy. Imaris Viewer (Oxford Instruments) was used to analyze images.

### Material availability

All materials are available upon request to keppler@mvp.lmu.de. This includes chicken anti-human SAMHD1 monoclonal antibody and proviral constructs pHIV-1* GFP, pHIV-1* BFP and pX4 HIVivo. These proviruses will also be made available through the National Institutes of Health AIDS Reagent Program. pTwist KI-Template GFP and pTwist KI-Template GFP–SAMHD1 are available from Addgene (plasmids 177988 and 177987, respectively).

### Reporting Summary

Further information is available in the Nature Research Reporting Summary linked to this article.

## Online content

Any methods, additional references, Nature Research reporting summaries, source data, extended data, supplementary information, acknowledgements, peer review information; details of author contributions and competing interests; and statements of data and code availability are available at 10.1038/s41592-021-01328-8.

## Supplementary information

Supplementary Information Flow cytometry gating strategy of HIV-1 challenged T cells. **a**, Example of the flow cytometry gating strategy in experiments with HIV-1 GFP infection. **b**, Example of the flow cytometry gating strategy in experiments with HIV-1 fusion.

Reporting Summary

Supplementary Table 1 Worksheet tab 1 ‘gRNAs’, sequence and more detailed information of the gRNAs used. Worksheet tab 2 ‘Primer’, sequence and more detailed information of the primer used. Worksheet tab 3 ‘KI templates’, sequence and more detailed information of the knock in templates used. Worksheet tab 4 ‘Off-Target loci’, sequence and more detailed information of the considered off-target sites

## Data Availability

The data in this paper are shown in the main figures and Extended Data figures. Additional information is available as Source Data Files for Figs. 1–4, Extended Data Figs. 1, 3, 6 and 7 as well as Supplementary Data. Source data are provided with this paper.

## Source data

Source Data Fig. 1 Statistical Source Data.
Source Data Fig. 2 Statistical Source Data, unmodified gels.
Source Data Fig. 3 Statistical Source Data, unmodified gels.
Source Data Fig. 4 Statistical Source Data.
Source Data Extended Data Fig. 1 Statistical Source Data.
Source Data Extended Data Fig. 3 Statistical Source Data.
Source Data Extended Data Fig. 6 Statistical Source Data.
Source Data Extended Data Fig. 7 Statistical Source Data.
Source Data Figs. 2, 3 and 5 and Extended Data Figs. 2, 4, 8 and 9 Uncropped gels for multiple figures contained in one PDF.

## References

[1] Pan X, Baldauf HM, Keppler OT, Fackler OT (2013). Restrictions to HIV-1 replication in resting CD4^+^ T lymphocytes. Cell Res..

[2] Berger, A. et al. SAMHD1-deficient CD14^+^ cells from individuals with Aicardi–Goutières syndrome are highly susceptible to HIV-1 infection. *PLoS Pathog*. 10.1371/journal.ppat.1002425 (2011). 10.1371/journal.ppat.1002425 PMC3234228 22174685

[3] Baldauf HM (2012). SAMHD1 restricts HIV-1 infection in resting CD4^+^ T cells. Nat. Med..

[4] Descours, B. et al. SAMHD1 restricts HIV-1 reverse transcription in quiescent CD4^+^ T-cells. *Retrovirology*10.1186/1742-4690-9-87 (2012). 10.1186/1742-4690-9-87 PMC3494655 23092122

[5] Hrecka K (2011). Vpx relieves inhibition of HIV-1 infection of macrophages mediated by the SAMHD1 protein. Nature.

[6] Laguette N (2011). SAMHD1 is the dendritic- and myeloid-cell-specific HIV-1 restriction factor counteracted by Vpx. Nature.

[7] Baldauf HM (2017). Vpx overcomes a SAMHD1-independent block to HIV reverse transcription that is specific to resting CD4 T cells. Proc. Natl Acad. Sci. USA.

[8] Liang G (2019). Membrane metalloprotease TRABD2A restricts HIV-1 progeny production in resting CD4^+^ T cells by degrading viral Gag polyprotein. Nat. Immunol..

[9] Hultquist JF (2016). A Cas9 ribonucleoprotein platform for functional genetic studies of HIV-host interactions in primary human T cells. Cell Rep..

[10] Hultquist, J. F. et al. CRISPR–Cas9 genome engineering of primary CD4^+^ T cells for the interrogation of HIV–host factor interactions. *Nat. Protoc*. 10.1038/s41596-018-0069-7 (2019). 10.1038/s41596-018-0069-7 PMC6637941 30559373

[11] Schumann K (2015). Generation of knock-in primary human T cells using Cas9 ribonucleoproteins. Proc. Natl Acad. Sci. USA.

[12] Seki A, Rutz S (2018). Optimized RNP transfection for highly efficient CRISPR/Cas9-mediated gene knockout in primary T cells. J. Exp. Med..

[13] Doudna JA, Charpentier E (2014). The new frontier of genome engineering with CRISPR-Cas9. Science.

[14] Linder A (2020). CARD8 inflammasome activation triggers pyroptosis in human T cells. EMBO J..

[15] Trinité B (2013). An HIV-1 replication pathway utilizing reverse transcription products that fail to integrate. J. Virol..

[16] Trinité B, Chan CN, Lee CS, Levy DN (2016). HIV-1 Vpr- and reverse transcription-induced apoptosis in resting peripheral blood CD4 T cells and protection by common γ-chain cytokines. J. Virol..

[17] Seya T, Atkinson JP (1989). Functional properties of membrane cofactor protein of complement. Biochem. J..

[18] Cattaneo R (2004). Four viruses, two bacteria, and one receptor: membrane cofactor protein (CD46) as pathogens’ magnet. J. Virol..

[19] Mathieson T (2018). Systematic analysis of protein turnover in primary cells. Nat. Commun..

[20] Feng Y, Broder CC, Kennedy PE, Berger EA (1996). HIV-1 entry cofactor: functional cDNA cloning of a seven-transmembrane, G protein-coupled receptor. Science.

[21] Cavrois M, De Noronha C, Greene WC (2002). A sensitive and specific enzyme-based assay detecting HIV-1 virion fusion in primary T lymphocytes. Nat. Biotechnol..

[22] Venzke S, Michel N, Allespach I, Fackler OT, Keppler OT (2006). Expression of Nef downregulates CXCR4, the major coreceptor of human immunodeficiency virus, from the surfaces of target cells and thereby enhances resistance to superinfection. J. Virol..

[23] Liu Y (2019). Proteomic profiling of HIV-1 infection of human CD4^+^ T cells identifies PSGL-1 as an HIV restriction factor. Nat. Microbiol..

[24] Fu Y (2020). PSGL-1 restricts HIV-1 infectivity by blocking virus particle attachment to target cells. Proc. Natl Acad. Sci. USA.

[25] Kane M (2013). MX2 is an interferon-induced inhibitor of HIV-1 infection. Nature.

[26] Bejarano DA (2019). HIV-1 nuclear import in macrophages is regulated by CPSF6-capsid interactions at the nuclear pore complex. eLife.

[27] Liu, Y. et al. PSGL-1 inhibits HIV-1 infection by restricting actin dynamics and sequestering HIV envelope proteins. *Cell Discov*. 10.1038/s41421-020-0184-9 (2020). 10.1038/s41421-020-0184-9 PMC7400672 32802403

[28] Kane M (2018). Nuclear pore heterogeneity influences HIV-1 infection and the antiviral activity of MX2. eLife.

[29] Sowd GA (2016). A critical role for alternative polyadenylation factor CPSF6 in targeting HIV-1 integration to transcriptionally active chromatin. Proc. Natl Acad. Sci. USA.

[30] Roth TL (2018). Reprogramming human T cell function and specificity with non-viral genome targeting. Nature.

[31] Govender U, Corre B, Bourdache Y, Pellegrini S, Michel F (2017). Type I interferon-enhanced IL-10 expression in human CD4 T cells is regulated by STAT3, STAT2, and BATF transcription factors. J. Leukoc. Biol..

[32] Sternberg SH, Redding S, Jinek M, Greene EC, Doudna JA (2014). DNA interrogation by the CRISPR RNA-guided endonuclease Cas9. Nature.

[33] Kawabe, T., Yi, J. & Sprent, J. Homeostasis of naive and memory T lymphocytes. *Cold Spring Harb. Perspect. Biol*. 10.1101/CSHPERSPECT.A037879 (2021). 10.1101/cshperspect.a037879 PMC8411951 33753403

[34] Pace MJ (2012). Directly infected resting CD4^+^ T cells can produce HIV Gag without spreading infection in a model of HIV latency. PLoS Pathog..

[35] Swiggard WJ (2005). Human immunodeficiency virus type 1 can establish latent infection in resting CD4^+^ T cells in the absence of activating stimuli. J. Virol..

[36] Siliciano JD, Siliciano RF (2021). Low inducibility of latent human immunodeficiency virus type 1 proviruses as a major barrier to cure. J. Infect. Dis..

[37] Grivel JC, Margolis L (2009). Use of human tissue explants to study human infectious agents. Nat. Protoc..

[38] Zutz A (2020). SERINC5 is an unconventional HIV restriction factor that is upregulated during myeloid cell differentiation. J. Innate Immun..

[39] Brinkman EK, Chen T, Amendola M, Van Steensel B (2014). Easy quantitative assessment of genome editing by sequence trace decomposition. Nucleic Acids Res..

[40] Schmid-Burgk JL (2014). OutKnocker: a web tool for rapid and simple genotyping of designer nuclease edited cell lines. Genome Res..

[41] Kim KH, Sederstrom JM (2015). Assaying cell cycle status using flow cytometry. Curr. Protoc. Mol. Biol..

[42] Levy DN, Aldrovandi GM, Kutsch O, Shaw GM (2004). Dynamics of HIV-1 recombination in its natural target cells. Proc. Natl Acad. Sci. USA.

[43] Albanese, M. et al. Epstein–Barr virus microRNAs reduce immune surveillance by virus-specific CD8^+^ T cells. *Proc. Natl Acad. Sci. USA*10.1073/pnas.1605884113 (2016). 10.1073/pnas.1605884113 PMC5081573 27698133

[44] Horwitz JA (2017). Non-neutralizing antibodies alter the course of HIV-1 infection in vivo. Cell.

[45] Geuenich, S. et al. Aqueous extracts from peppermint, sage and lemon balm leaves display potent anti-HIV-1 activity by increasing the virion density. *Retrovirology*10.1186/1742-4690-5-27 (2008). 10.1186/1742-4690-5-27 PMC2288616 18355409

[46] Braun E (2019). Guanylate-binding proteins 2 and 5 exert broad antiviral activity by inhibiting furin-mediated processing of viral envelope proteins. Cell Rep..

