# Supplementary material for: Rapid, efficient and activation-neutral gene editing of polyclonal primary human resting CD4+ T cells allows complex functional analyses
Source: Nat Methods. 2021 Dec 23;19(1):81–9. doi: 10.1038/s41592-021-01328-8 (PMC8748193; doi:10.1038/s41592-021-01328-8)
Supplement: Supplementary file 1 — Flow cytometry gating strategy of HIV-1 challenged T cells. a, Example of the flow cytometry gating strategy in experiments with HIV-1 GFP infection. b, Example of the flow cytometry gating strategy in experiments with HIV-1 fusion. [file 41592_2021_1328_MOESM1_ESM.pdf]

nature portfolio

https://doi.org/10.1038/s41592-021-01328-8

Supplementary information

Rapid, efficient and activation-neutral gene editing of polyclonal primary human resting CD4$^+$ T cells allows complex functional analyses

In the format provided by the authors and unedited

a

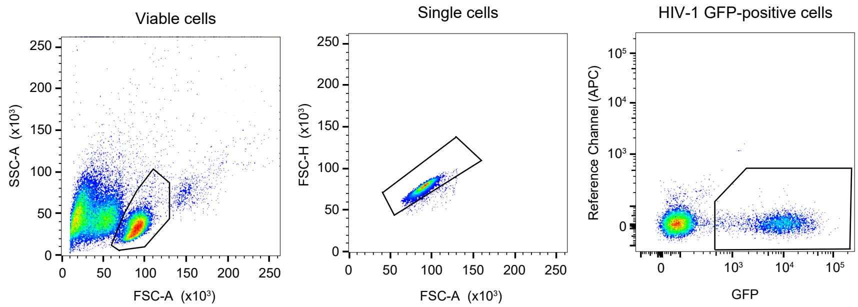

b

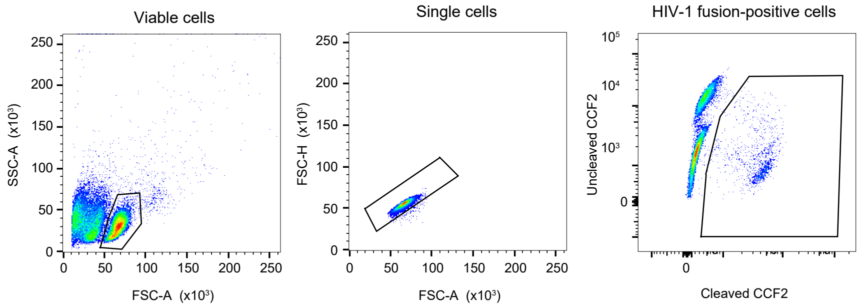

Supplementary Fig. 1

a b c

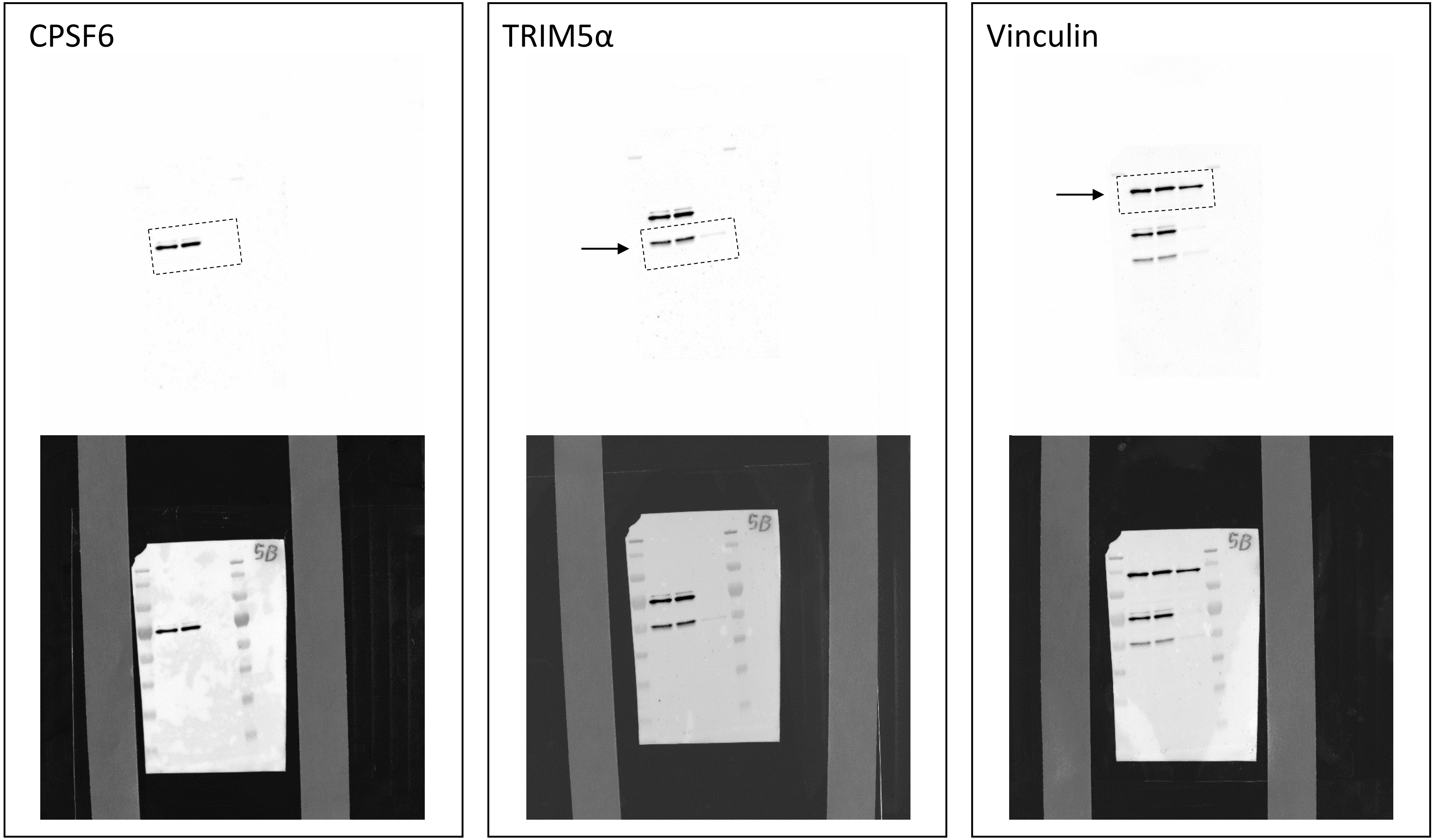

Supplementary Fig. 2

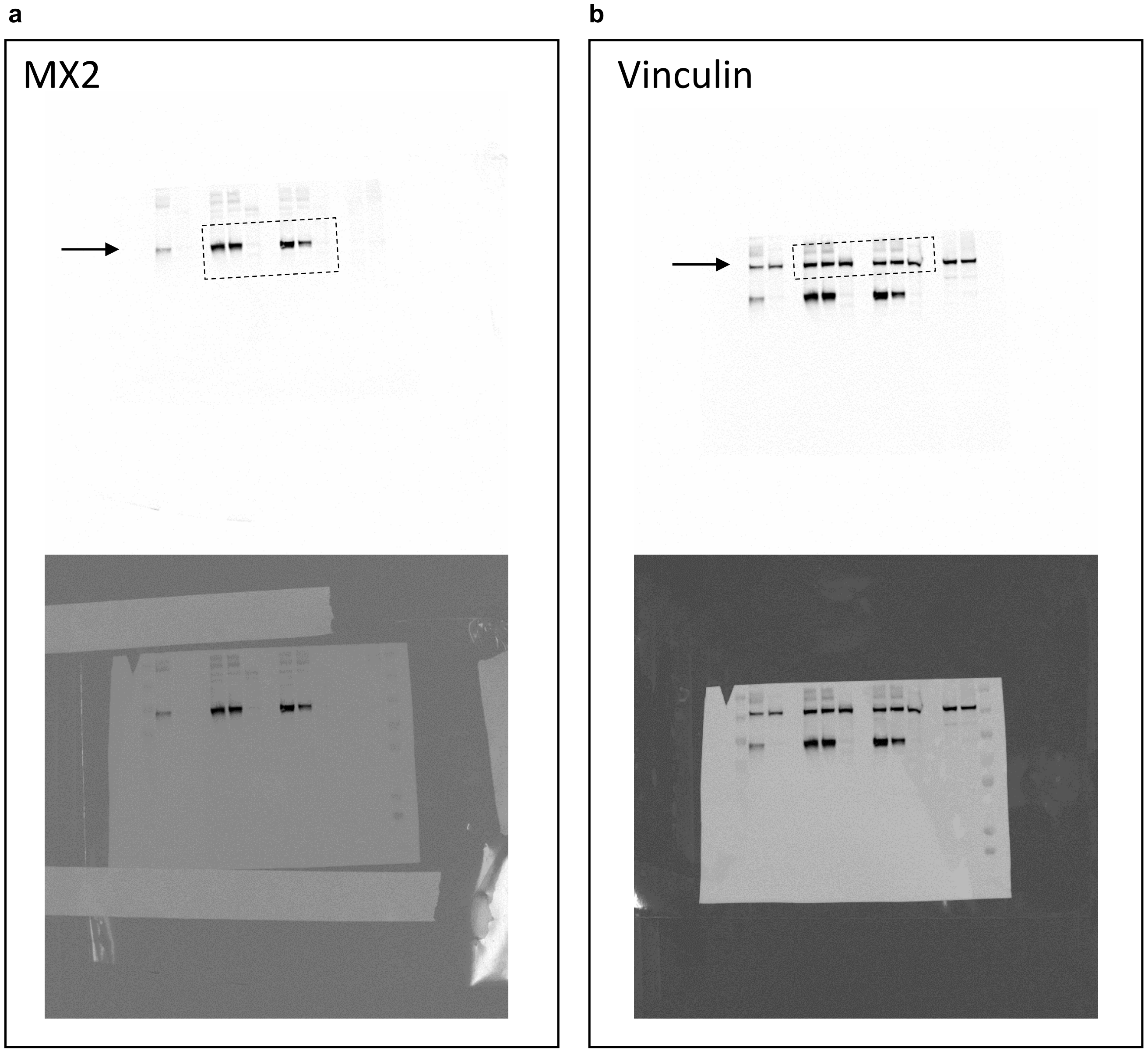

Supplementary Fig. 3

a

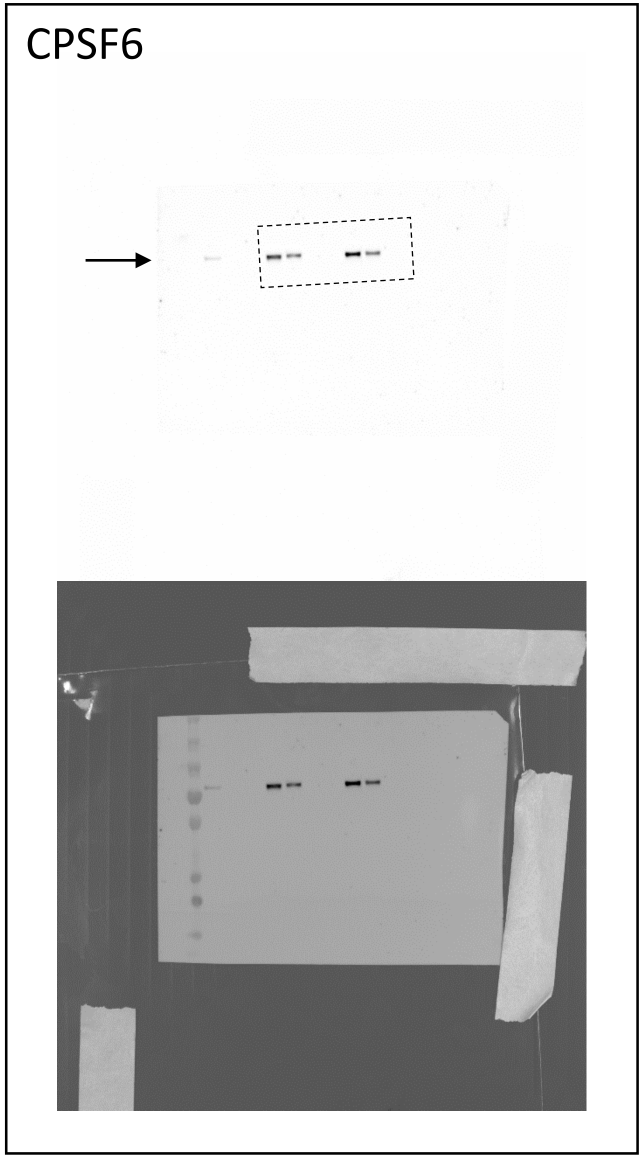

b

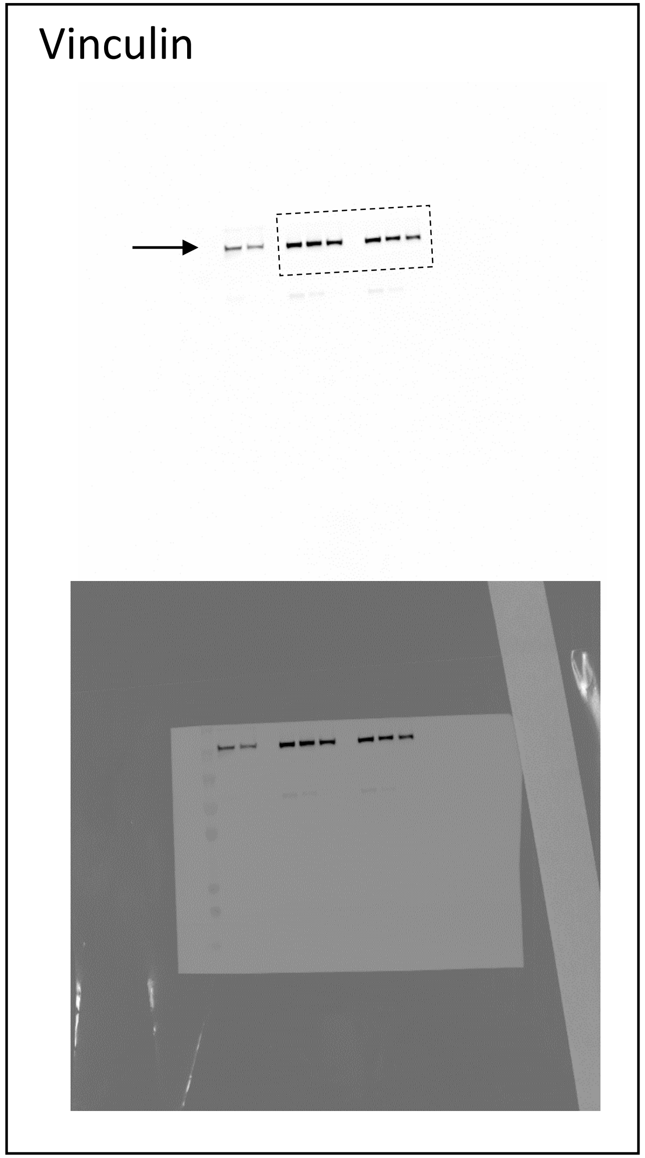

Supplementary Fig. 4

a

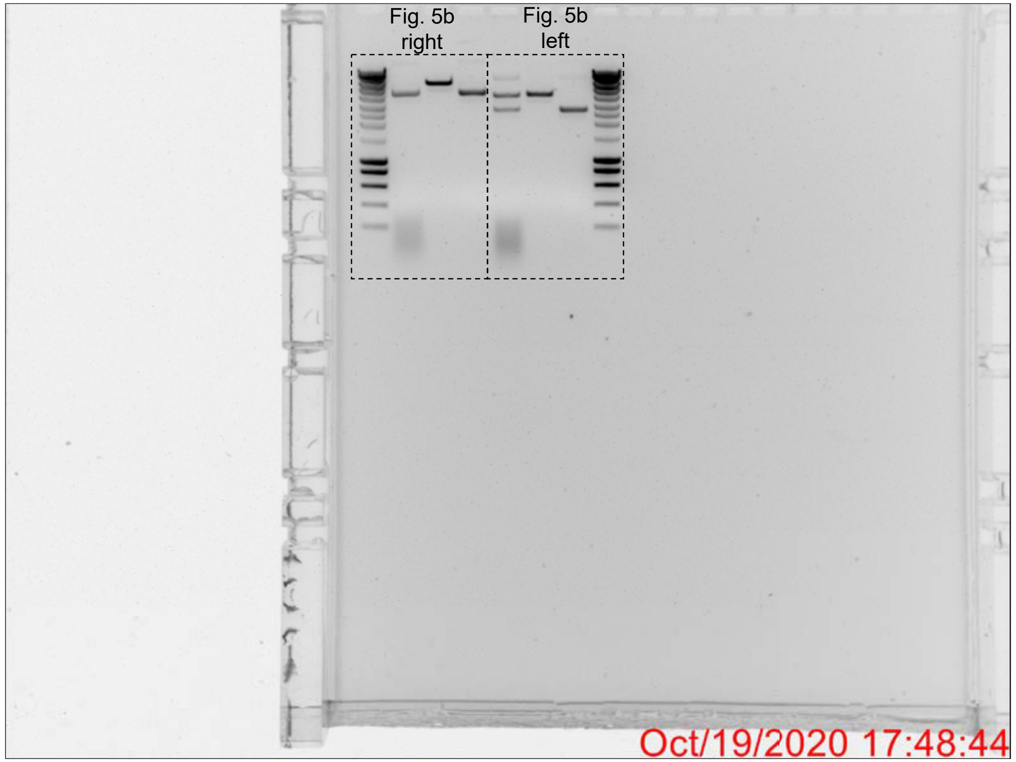

Supplementary Fig. 5

a

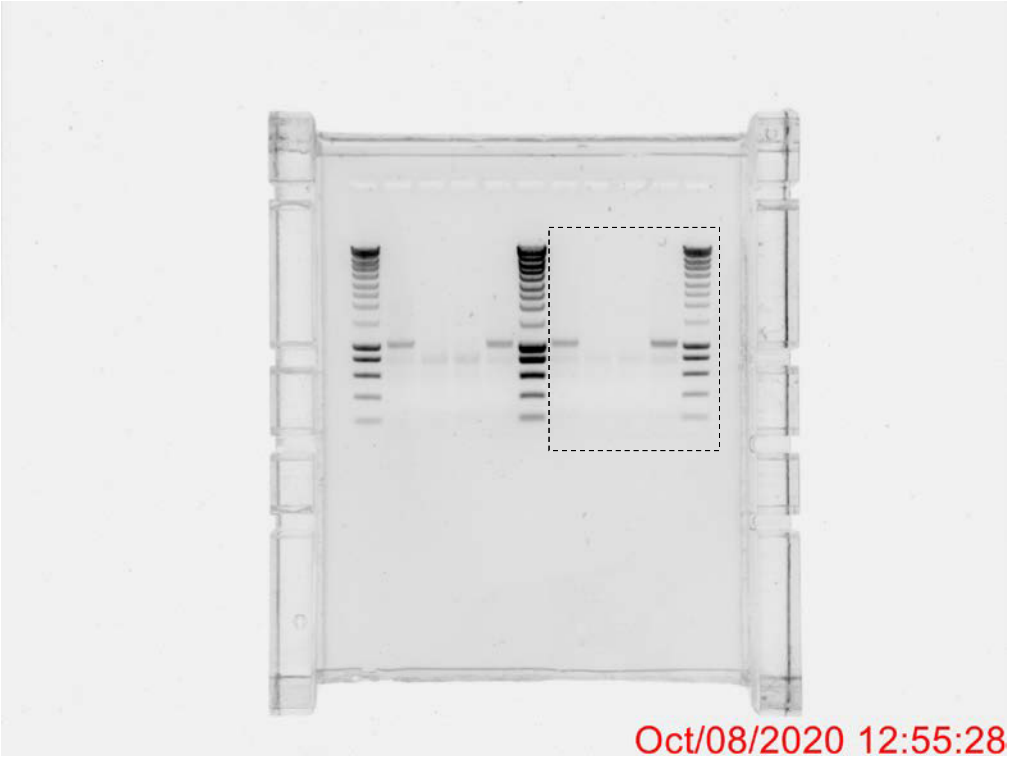

b

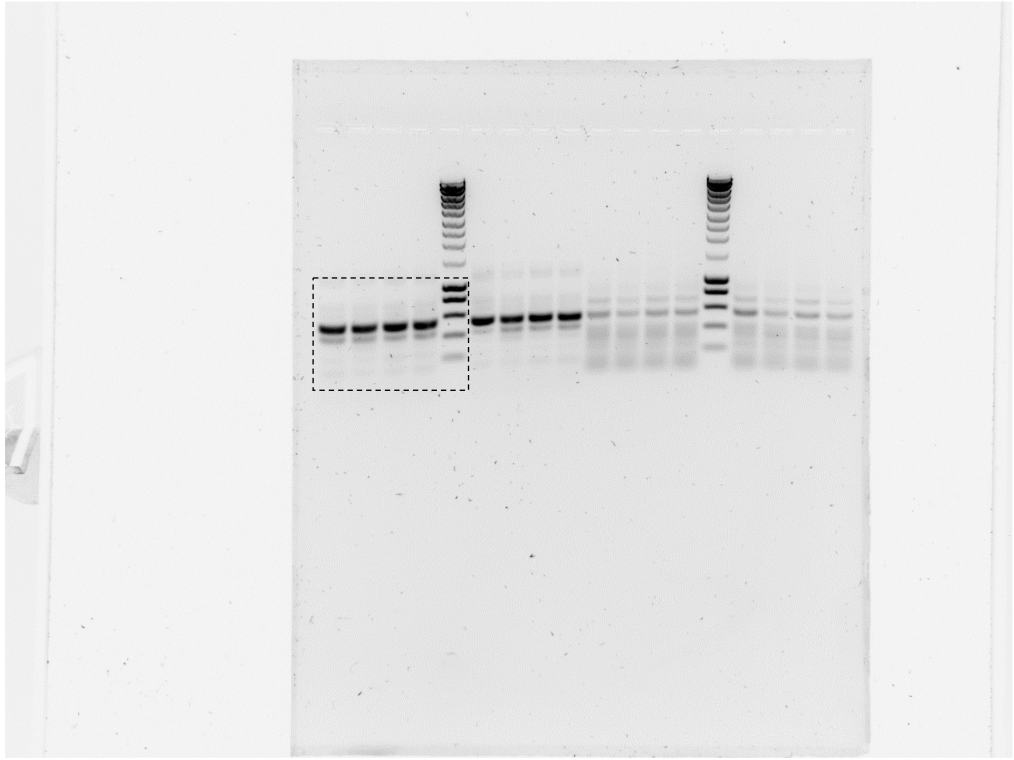

Supplementary Fig. 6

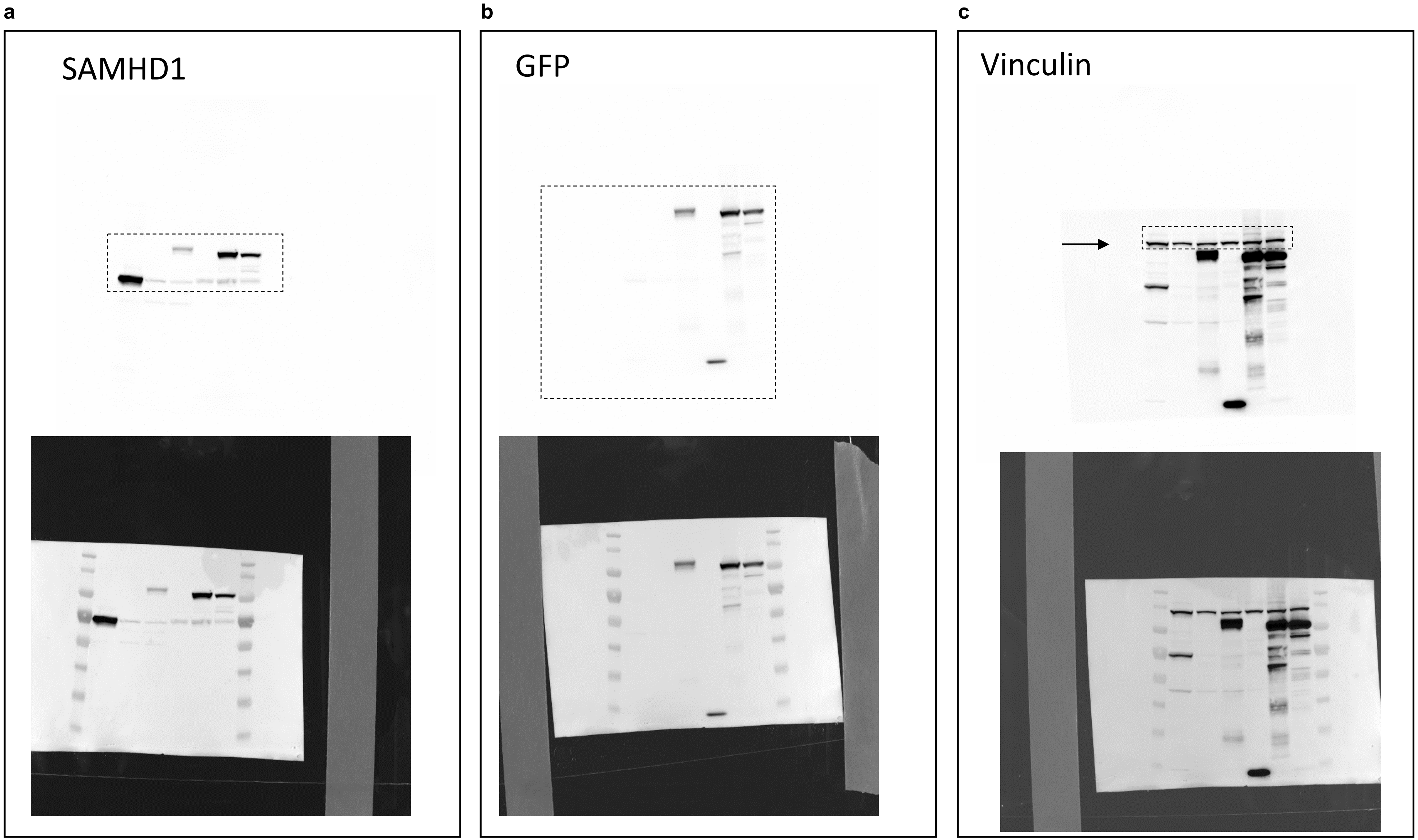

Supplementary Fig. 7

a

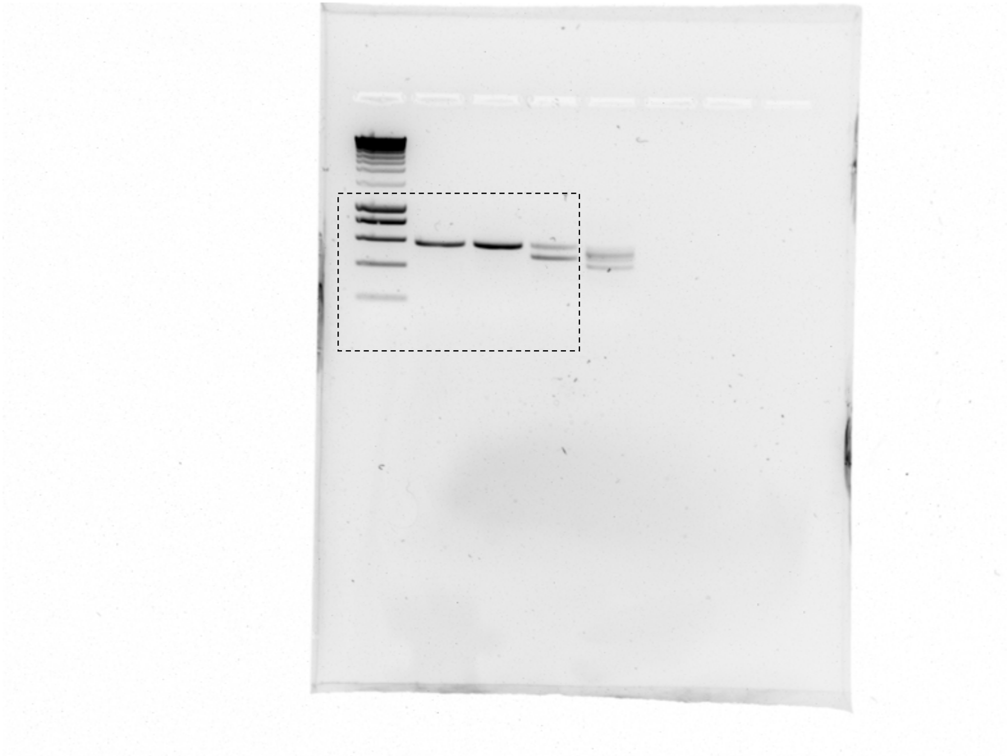

Supplementary Fig. 8

a

SAMHD1

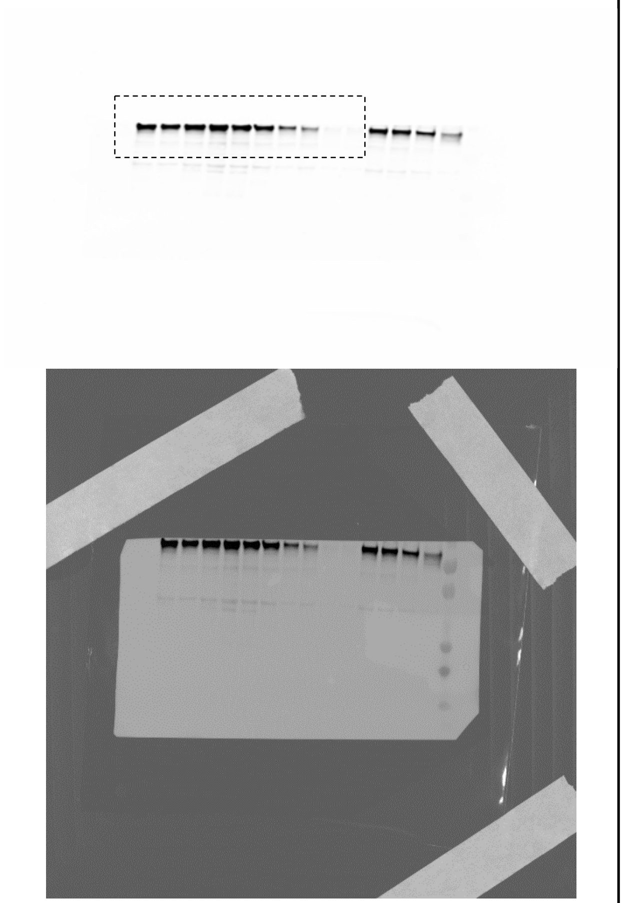

b

Vinculin

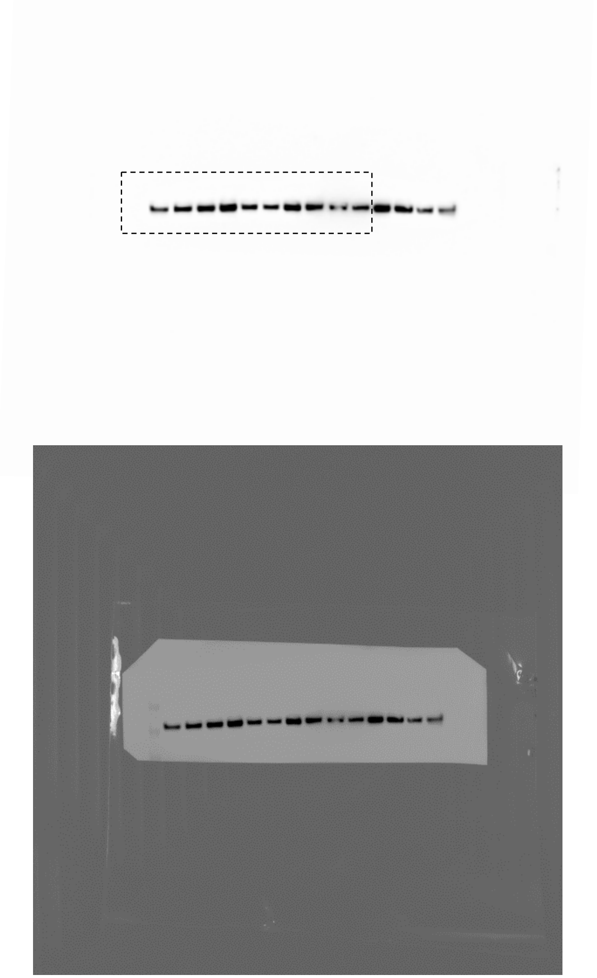

Supplementary Fig. 9

a

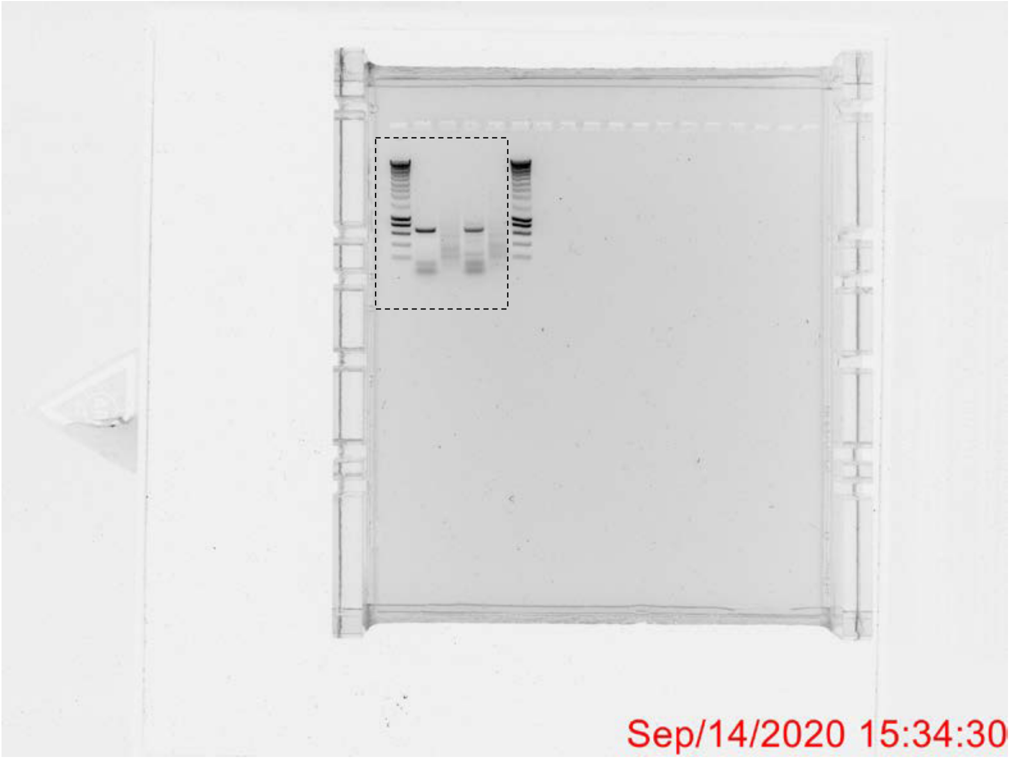

b

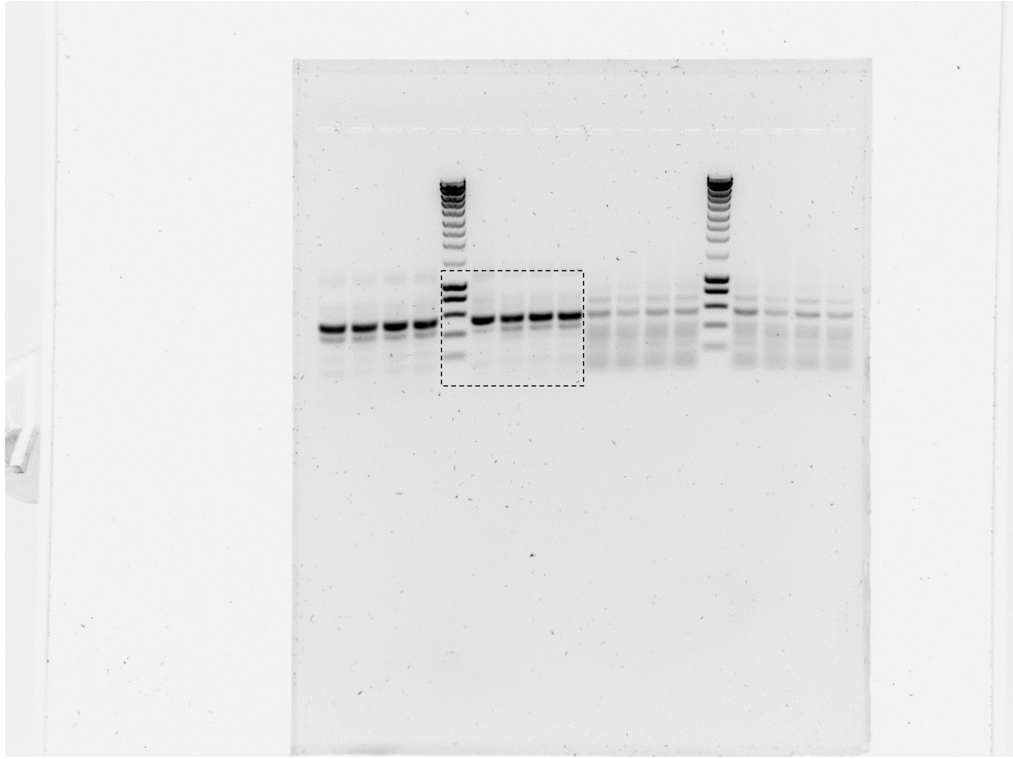

Supplementary Fig. 10

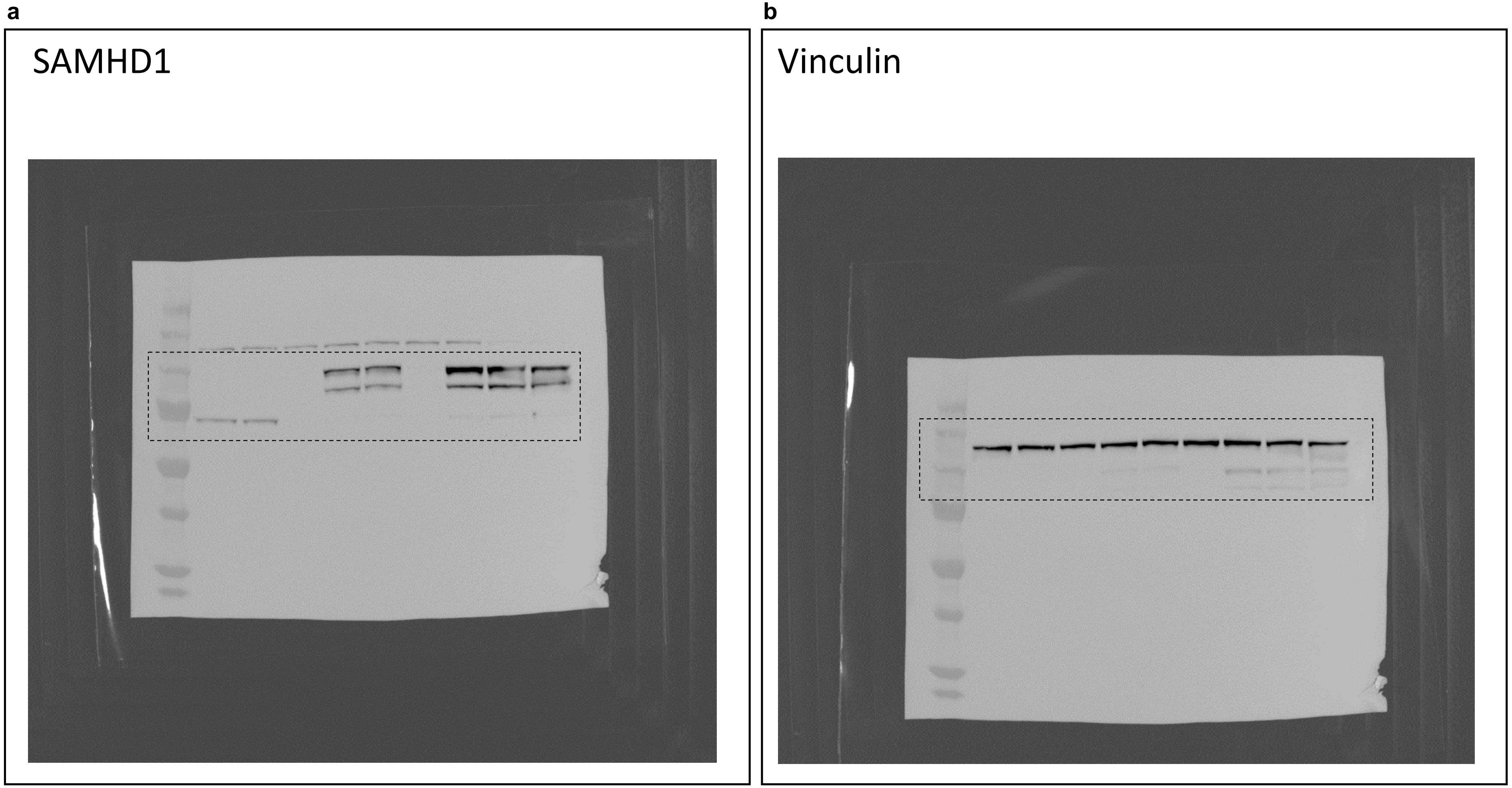

Supplementary Fig. 11
