# Supplementary material for: Rapid, efficient and activation-neutral gene editing of polyclonal primary human resting CD4+ T cells allows complex functional analyses
Source: Nat Methods. 2021 Dec 23;19(1):81–9. doi: 10.1038/s41592-021-01328-8 (PMC8748193; doi:10.1038/s41592-021-01328-8)
Supplement: Supplementary file 2 — Reporting Summary [file 41592_2021_1328_MOESM2_ESM.pdf]

nature research

Corresponding author(s): Oliver T. Keppler, Manuel Albanese

Last updated by author(s): 07.10.2021

Reporting Summary

Nature Research wishes to improve the reproducibility of the work that we publish. This form provides structure for consistency and transparency in reporting. For further information on Nature Research policies, see our Editorial Policies and the Editorial Policy Checklist.

Statistics

For all statistical analyses, confirm that the following items are present in the figure legend, table legend, main text, or Methods section.

n/a	Confirmed	
☐	☒	The exact sample size (*n*) for each experimental group/condition, given as a discrete number and unit of measurement
☐	☒	A statement on whether measurements were taken from distinct samples or whether the same sample was measured repeatedly
☐	☒	The statistical test(s) used AND whether they are one- or two-sided<br>*Only common tests should be described solely by name; describe more complex techniques in the Methods section.*
☒	☐	A description of all covariates tested
☐	☒	A description of any assumptions or corrections, such as tests of normality and adjustment for multiple comparisons
☐	☒	A full description of the statistical parameters including central tendency (e.g. means) or other basic estimates (e.g. regression coefficient) AND variation (e.g. standard deviation) or associated estimates of uncertainty (e.g. confidence intervals)
☐	☒	For null hypothesis testing, the test statistic (e.g. *F*, *t*, *r*) with confidence intervals, effect sizes, degrees of freedom and *P* value noted<br>*Give P values as exact values whenever suitable.*
☒	☐	For Bayesian analysis, information on the choice of priors and Markov chain Monte Carlo settings
☒	☐	For hierarchical and complex designs, identification of the appropriate level for tests and full reporting of outcomes
☒	☐	Estimates of effect sizes (e.g. Cohen's *d*, Pearson's *r*), indicating how they were calculated

Our web collection on statistics for biologists contains articles on many of the points above.

Software and code

Policy information about availability of computer code

Data collection	Compass for simple westernblot (proteinsimple) [V5.0.0], NIS-Elements (Nikon), FUSION FX Edge (Vilber)[V18.02], VisionWorks (Analytik Jena) [V4.15], BD FACS Suite (BD)[V1.2.1].
Data analysis	FlowJo V10 for flow cytometry analysis; GraphPad Prism 7 or 8 for statistical calculation and graph design; TIDE (Tracking indels by decomposition; https://tide.nki.nl/) and Outknocker 2.0 (http://www.outknocker.org/outknocker2.htm) for analysis of sequencing results, Adobe Illustrator CS6, Compass for simple westernblot (proteinsimple)[V5.0.0], Snapgene 4.2.11, Imaris Viewer (Oxford Instruments)[V9.6.0]

For manuscripts utilizing custom algorithms or software that are central to the research but not yet described in published literature, software must be made available to editors and reviewers. We strongly encourage code deposition in a community repository (e.g. GitHub). See the Nature Research guidelines for submitting code & software for further information.

Data

Policy information about availability of data

All manuscripts must include a data availability statement. This statement should provide the following information, where applicable:

- Accession codes, unique identifiers, or web links for publicly available datasets
- A list of figures that have associated raw data
- A description of any restrictions on data availability

The data in this paper is shown in the main figures and Extended Data figures. Additional information is available as Source Data Files for Figures 1, 2, 3, and 4, Extended Data Figures 1, 3, 6, and 7 as well as Supplementary Data files.

Field-specific reporting

Please select the one below that is the best fit for your research. If you are not sure, read the appropriate sections before making your selection.

☒ Life sciences ☐ Behavioural & social sciences ☐ Ecological, evolutionary & environmental sciences

For a reference copy of the document with all sections, see nature.com/documents/nr-reporting-summary-flat.pdf

Life sciences study design

All studies must disclose on these points even when the disclosure is negative.

Sample size	No statistical tests were used to predetermine sample size.<br>Primary cells were isolated from different healthy blood donors whereby the cells of one donor count as one sample.
Data exclusions	no data were excluded
Replication	Number of donors "n" is indicated in the figure legends for each experiment. The experiments are performed with at least three different healthy blood donors if not other mentioned. Only biological replicates are shown.
Randomization	We received blood samples from anonymous healthy blood donors. No further randomization was applied as it is not relevant for the study.
Blinding	The investigators were neither blinded during data collection nor analysis as it is not relevant for the study.

Reporting for specific materials, systems and methods

We require information from authors about some types of materials, experimental systems and methods used in many studies. Here, indicate whether each material, system or method listed is relevant to your study. If you are not sure if a list item applies to your research, read the appropriate section before selecting a response.

Materials & experimental systems

n/a	Involved in the study	
☐	☒	Antibodies
☐	☒	Eukaryotic cell lines
☒	☐	Palaeontology and archaeology
☒	☐	Animals and other organisms
☐	☒	Human research participants
☒	☐	Clinical data
☒	☐	Dual use research of concern

Methods

n/a	Involved in the study	
☒	☐	ChIP-seq
☐	☒	Flow cytometry
☒	☐	MRI-based neuroimaging

Antibodies

Antibodies used

Flow cytometry and microscopy: α-CD46 (BV421 [Cat. No. 743776] and PE [Cat. No. 564252] clone E4.3, BD; APC [Cat. No. 352405] clone TRA-2-10, Biolegend), α-CXCR4 (BV421 [Cat. No. 562448]; PE-Cy.5[Cat. No. 555975] clone 12G5, BD; PE-Cy7 [Cat. No. 306514] clone 12G5, Biolegend), α-PSGL-1 (Alexa Fluor 647 [Cat. No. 328809] clone KPL-1, Biolegend; BV421 [Cat. No. 743478] clone KPL-1, BD), α-CD25 (BV421 [Cat. No. 562442] and APC [Cat. No. 555434] Clone M-A251, BD), α-CD69 (BV421 [Cat. No. 562884] and APC [Cat. No. 555533] Clone FN50, BD), a-CD4 (PE-Cy7 [Cat. No. 300512] clone RPA-T4, Biolegend; APC [Cat. No. 555349] clone RPA-T4, BD), a-CD38 (PE [Cat. No. 555460] clone HIT2, BD), a-HLA-DR (FITC [Cat. No. 347400] clone L243, BD), goat anti-Mouse IgG (H+L) (Alexa Fluor 647 [Cat. No. A-21236] polyclonal, Invitrogen), a-GFP (rabbit, polyclonal [code: PABG1-20], Chromoteck), goat anti-Rabbit IgG (H+L) (Alexa Fluor 647 [Cat. No. A-21245] polyclonal, Invitrogen). All antibodies for flow cytometry were used in a dilution of 1:50 or 1:100.
Immunoblot: α-SAMHD1 (proprietary chicken monoclonal Ab of the Keppler laboratory, clone H154, produced by Eurogentec) 1:4000, α-SAMHD1 (proprietary mouse monoclonal Ab of the Keppler laboratory, clone 1166E8-19D08-PG, produced by Eurogentec) 1:200, α-CPSF6 (Rabbit, polyclonal, Cell Signaling [Cat. No. 75168S]) 1:1000, α-Vinculin (mouse, hVIN-1, Sigma Aldrich [Cat. No. V9264]) 1:2000, α-MX2 (rabbit, polyclonal, Novus Biologicals [Cat. No. NBP1-81018]) 1:250, α-TRIM5a (rabbit, clone D6Z8L, Cell Signaling [Cat. No. 14326S]) 1:1000, α-SAMHD1 (mouse, clone OTI3F5, Origene [Cat. No. TA502024]) 1:250, a-GFP (rabbit polyclonal [code: PABG1-20] Chromoteck) 1:1000, The following secondary Ab HRP conjugated were used in a dilution of 1:10000: goat anti mouse IgG (Rat Adsorbed, polyclonal, Biorad [Cat. No. STAR77]), goat anti-chicken IgY (H&L, polyclonal, Abcam [Cat. No. ab6877]), goat-IgG anti-Rabbit IgG (H+L, polyclonal, Dianova [Cat. No. AFK-600])

Validation	All Antibodies used in this study were validated by the vendors and commonly used by the field. We additionally validate them using negative controls, KO cells for the protein of interest or isotype controls.<br>See here fore more informations:<br>https://www.biolegend.com/en-us/quality/product-development<br>https://www.thermofisher.com/de/de/home/life-science/antibodies.html.html<br>https://www.cellsignal.de/about-us/cst-antibody-validation-principles<br>https://www.abcam.com/primary-antibodies/a-guide-to-antibody-validation<br>https://www.bio-rad-antibodies.com/our-antibody-validation-principles.html<br>https://www.novusbio.com/reproducibility.html<br>https://www.sigmaaldrich.com/DE/en/technical-documents/technical-article/protein-biology/elisa/antibody-standard-validation<br>Proprietary Antibodies against SAMHD1 have been tested and titrated using SAMHD1 KO cells together with WT cells to assess the specificity.

Eukaryotic cell lines

Policy information about cell lines

Cell line source(s)	293T (DSMZ no. ACC 635); SupT1 (DSMZ no. ACC 140); LN18 (ATCC CRL-2610)
Authentication	Cell lines were authenticated by the commercial vendor. See : https://www.dsmz.de/ or https://www.atcc.org/.
Mycoplasma contamination	All cell lines were tested negative for mycoplasma contamination regularly
Commonly misidentified lines<br>(See ICLAC register)	No commonly misidentified cell lines were used

Human research participants

Policy information about studies involving human research participants

Population characteristics	Age between 20-55; male and female donors; >50 kg body weight; good general condition without any chronic infectious diseases (e.g. HIV, HCV)
Recruitment	Participants were recruited by the thrombocyte donation center of the LMU without any selection or interference by the investigators. Any person who met the criteria mentioned above was allowed to participate. Hence, we do not see any impact on the results due to the recruitment.
Ethics oversight	Ethics has been approved by the Ethics Committee of the LMU (Project No. 17-202 UE).

Note that full information on the approval of the study protocol must also be provided in the manuscript.

Flow Cytometry

Plots

Confirm that:

☒ The axis labels state the marker and fluorochrome used (e.g. CD4-FITC).

☒ The axis scales are clearly visible. Include numbers along axes only for bottom left plot of group (a 'group' is an analysis of identical markers).

☒ All plots are contour plots with outliers or pseudocolor plots.

☒ A numerical value for number of cells or percentage (with statistics) is provided.

Methodology

Sample preparation	Cells were collected, washed once with PBS and resuspended in 50 ul of stained solution, FACS buffer (PBS, 1%FBS, 2mM EDTA) and antibodies (Material and Methods), and kept 20 min at 4°C. After this time, cells were washed with FACS buffer and resuspended in 100 ul of FACS buffer. Cells were from healthy blood donors as described above.
Instrument	BD FACS Lyric
Software	Collecting software:BD FACS Suite<br>Analysing software: FlowJo V10
Cell population abundance	At least 10 000 cells were recorded for each gate of interest.
Gating strategy	If not other mentioned: 1. FSC/SSC: cells were gated on lymphocytes 2. FSC-A/FSC-H: single cells were gated 3. Boundaries for "positive" and "negative" cells were set according to the positive and negative controls in each experiment

☒ Tick this box to confirm that a figure exemplifying the gating strategy is provided in the Supplementary Information.
