# Supplementary material for: Rapid, efficient and activation-neutral gene editing of polyclonal primary human resting CD4+ T cells allows complex functional analyses
Source: Nat Methods. 2021 Dec 23;19(1):81–9. doi: 10.1038/s41592-021-01328-8 (PMC8748193; doi:10.1038/s41592-021-01328-8)
Supplement: Source Data Figs. 2, 3 and 5 and Extended Data Figs. 2, 4, 8 and 9 — Uncropped gels for multiple figures contained in one PDF. [file 41592_2021_1328_MOESM12_ESM.pdf]

Source data Figure 2

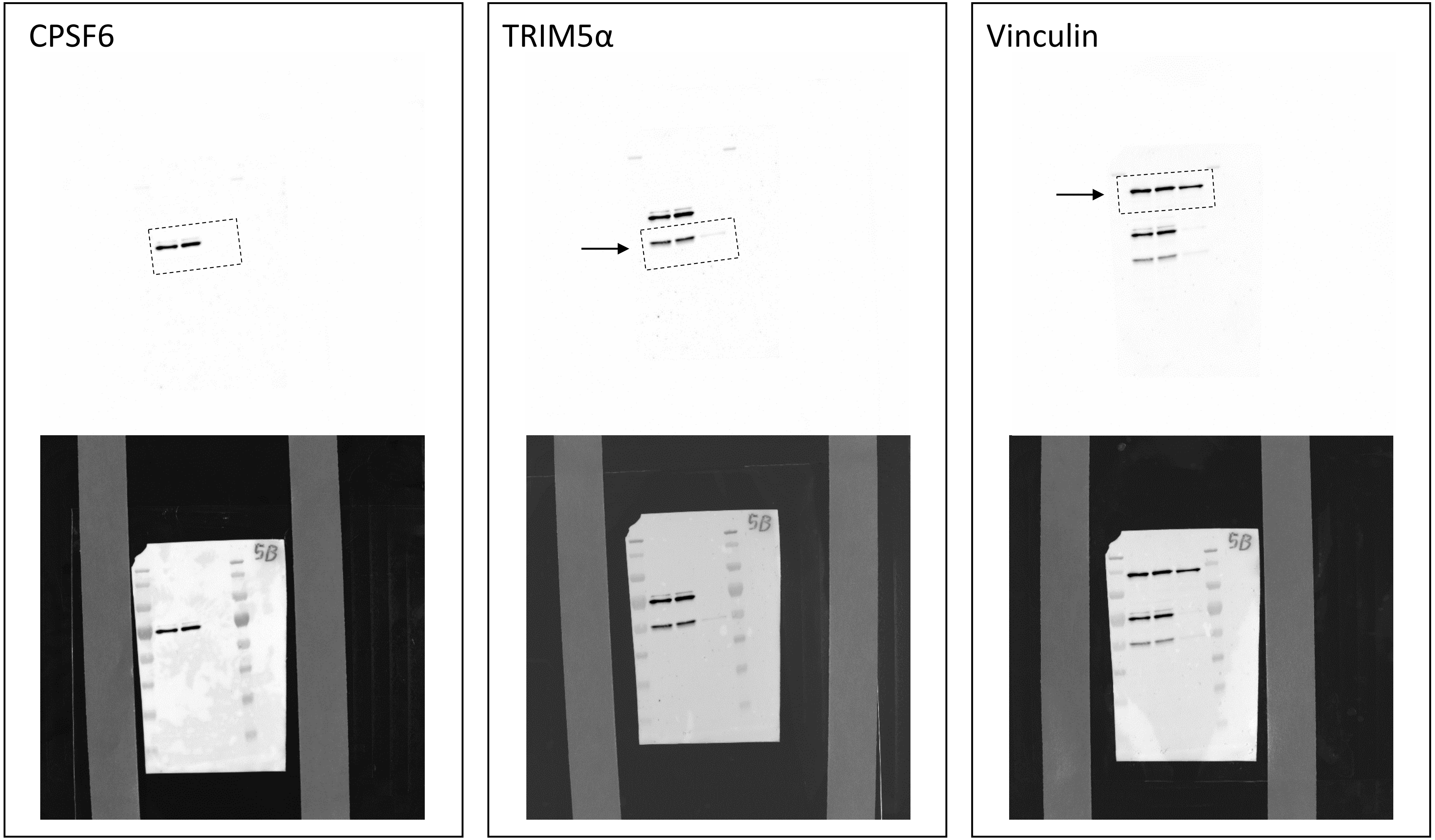

The same membrane was re-probed first against CPSF6, then TRIM5α, and finally against Vinculin.

Source data Figure 3i (top)

MX2

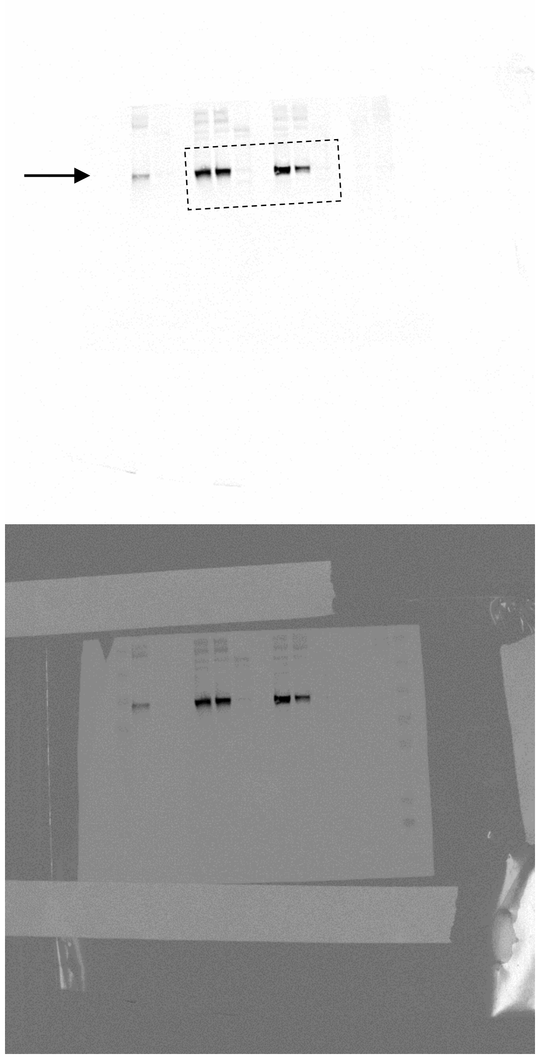

Vinculin

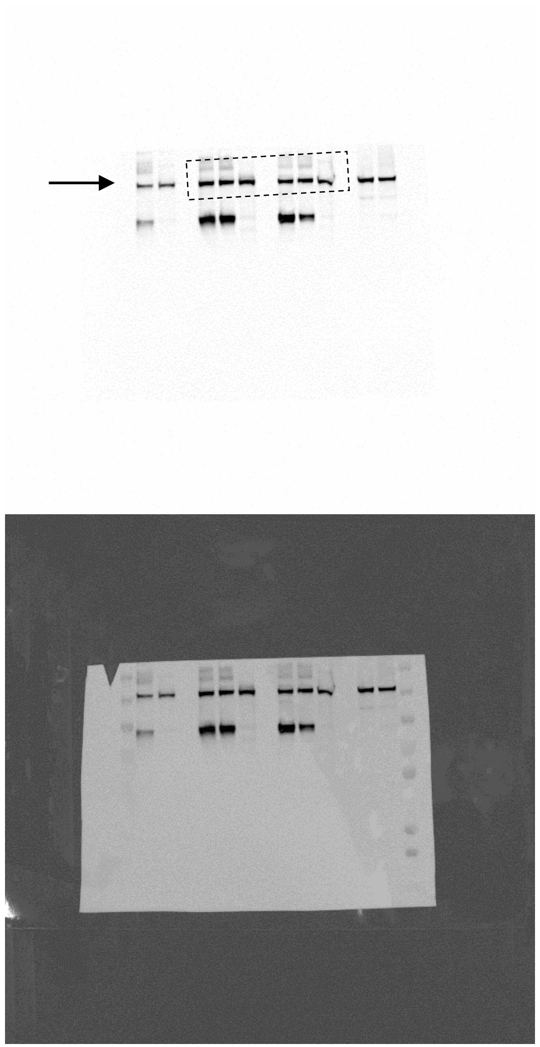

The same membrane was re-probed first against MX2, then against Vinculin.

Source data Figure 3i (bottom)

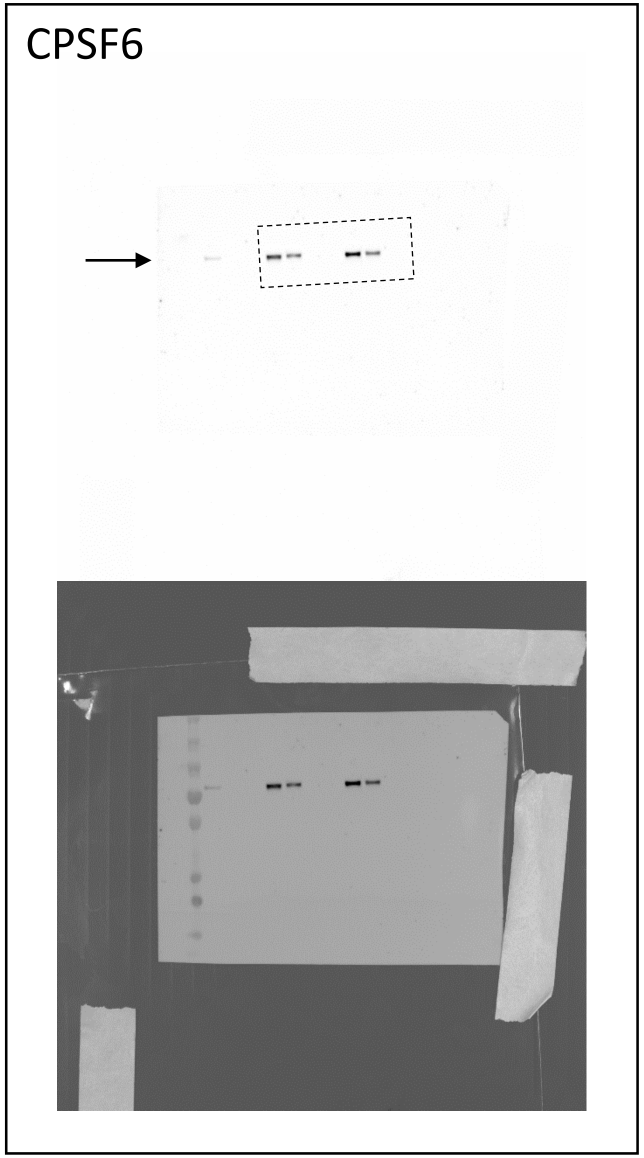

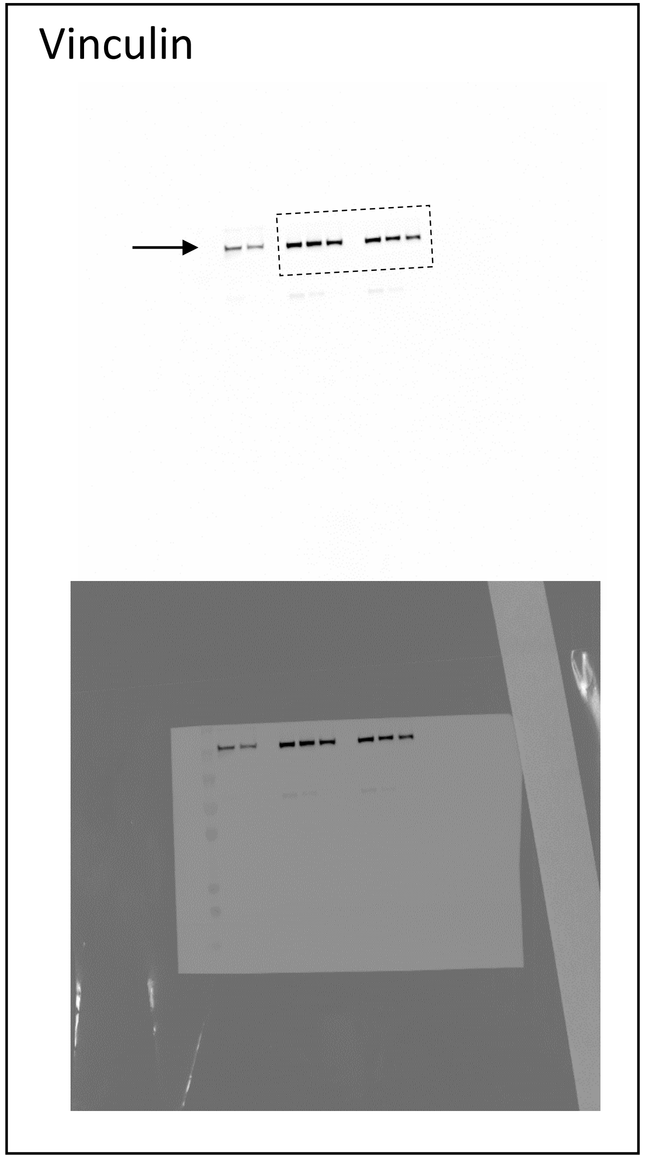

The same membrane was re-probed first against CPSF6, then against Vinculin.

Source data Figure 5b

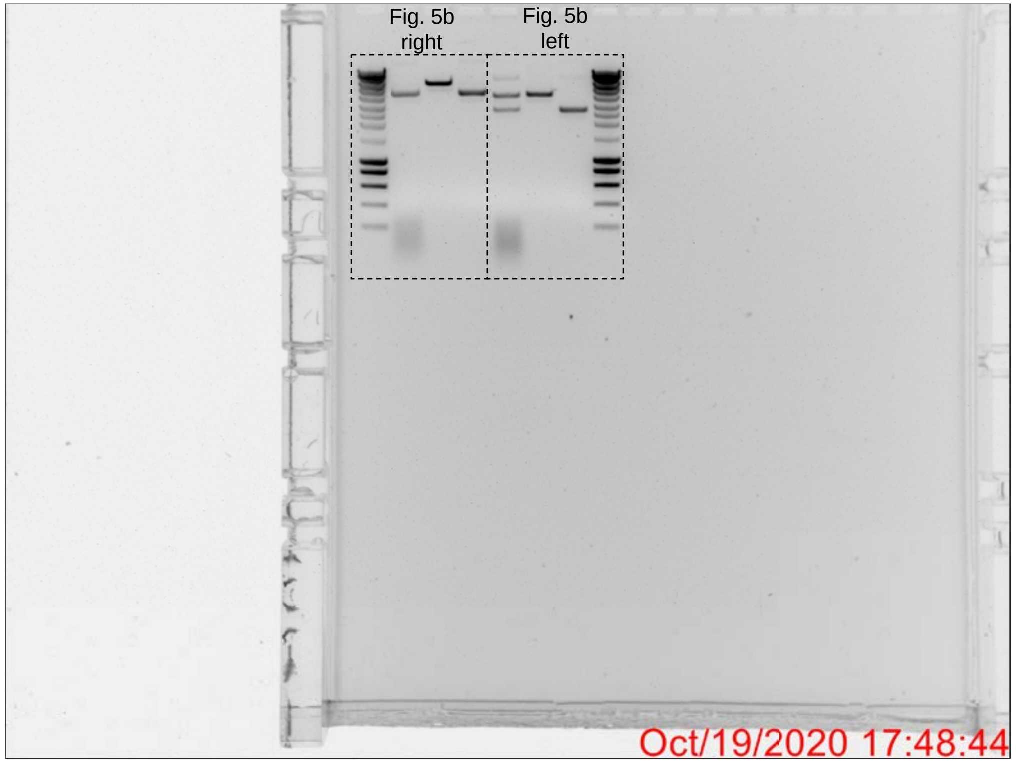

Source data Figure 5c

PCR SAMHD1 KI

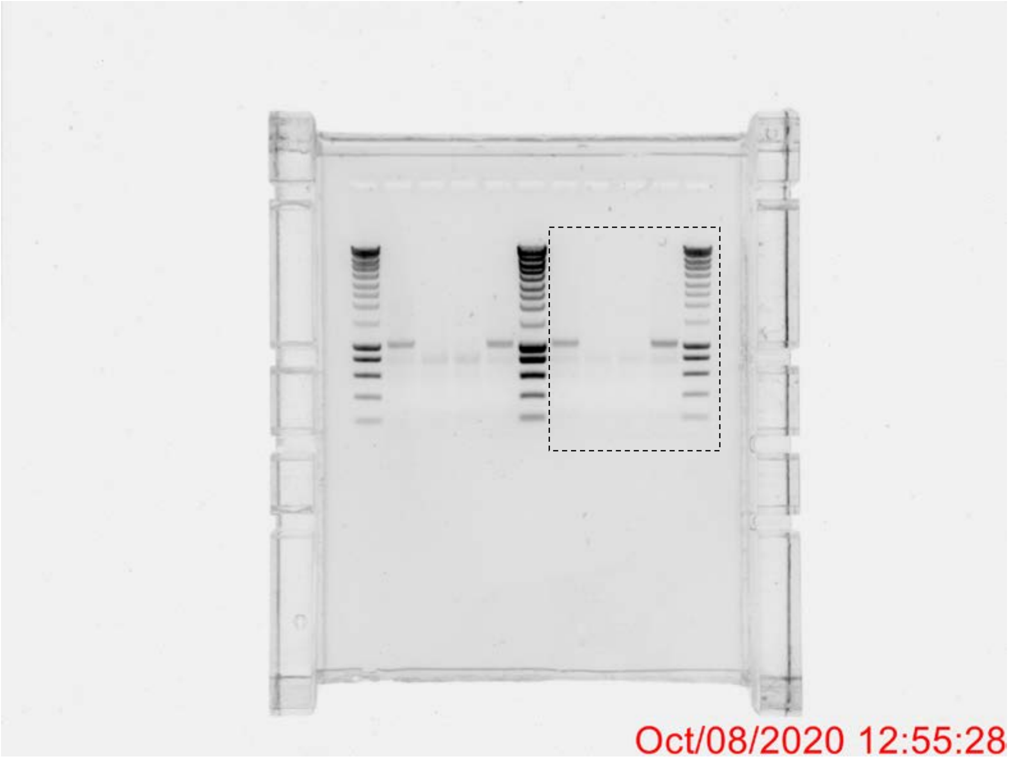

PCR *CD46 locus* (Loading control)

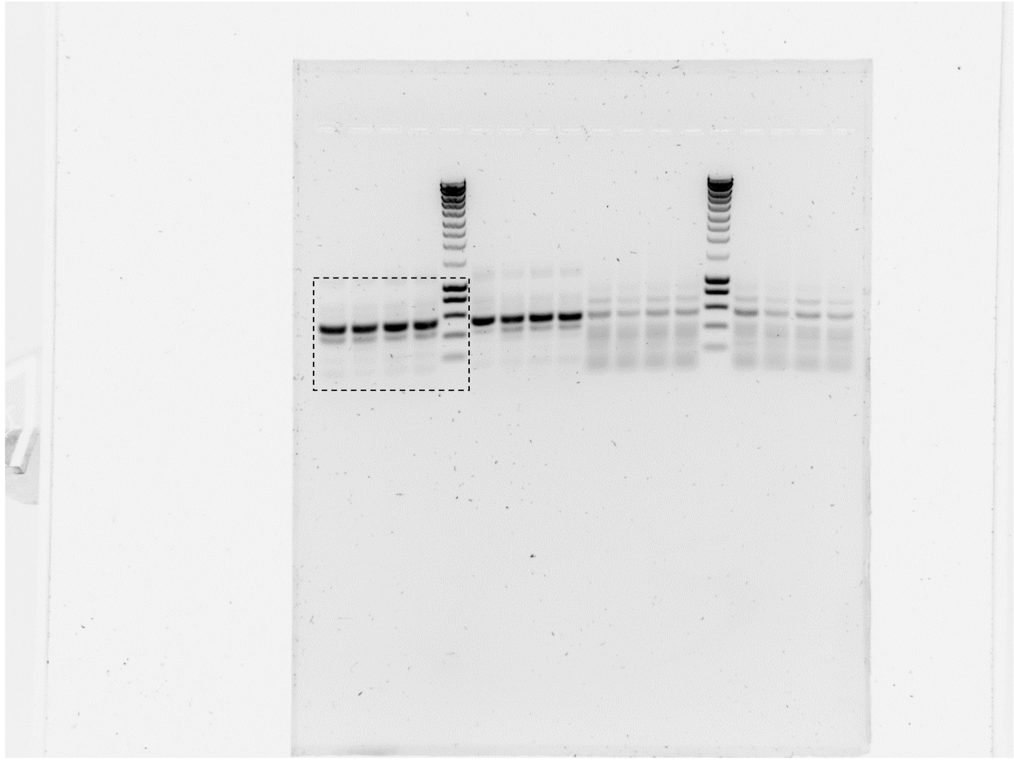

Source data Figure 5d

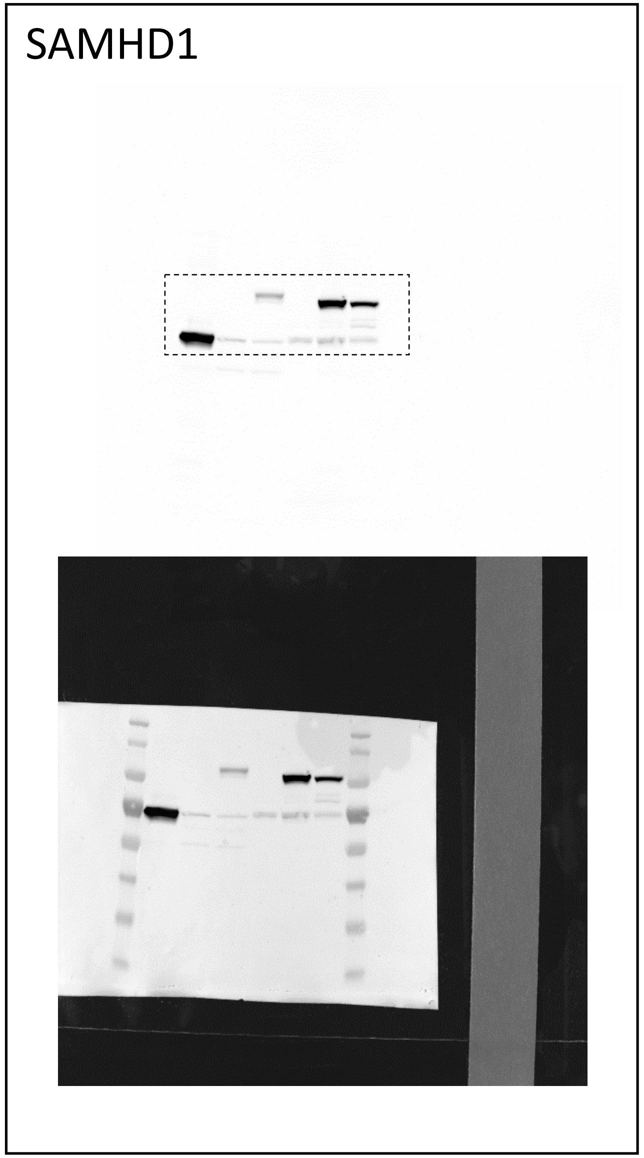

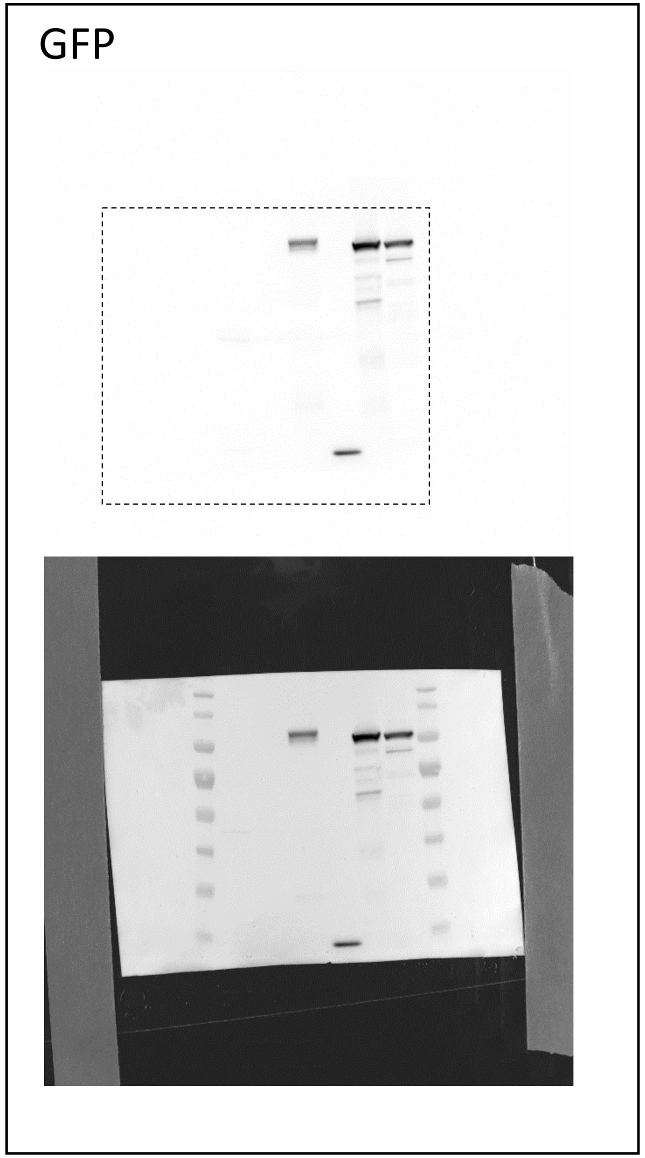

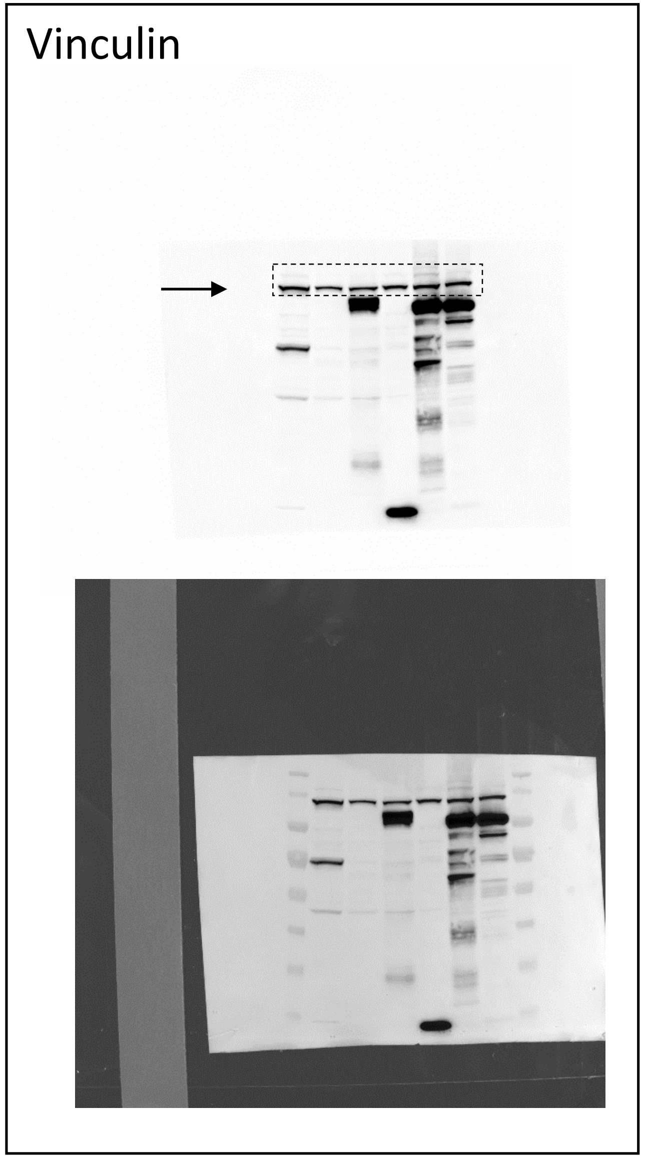

The same membrane was re-probed first against SAMHD1, then GFP and then against Vinculin.

Source Extended Data Figure 2c

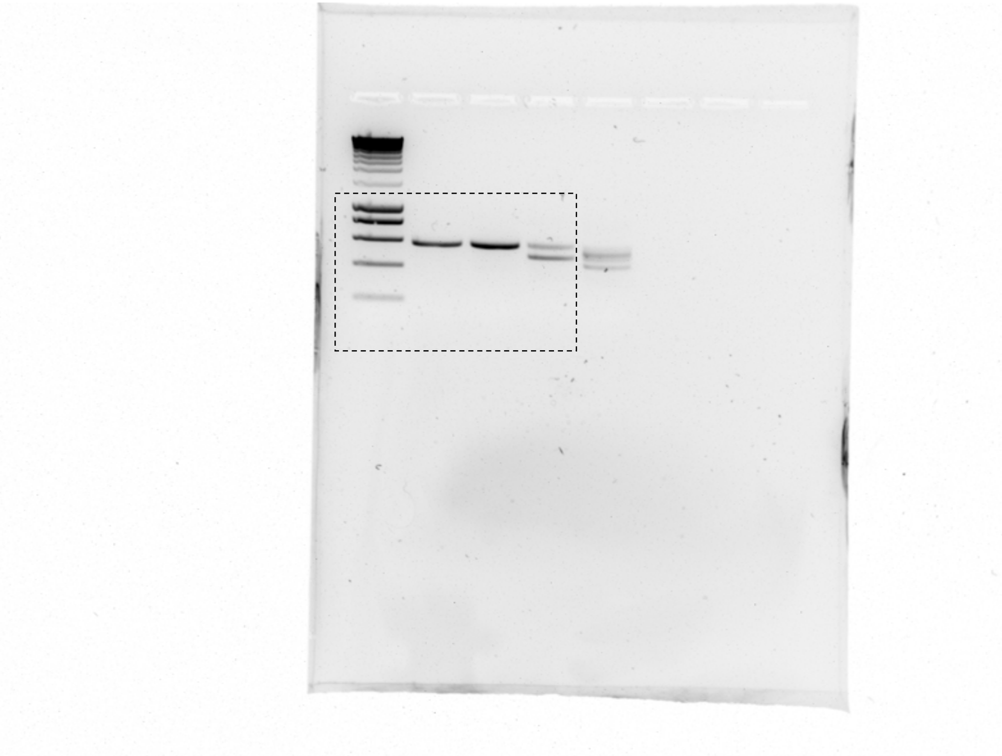

Source Extended Data Figure 4b

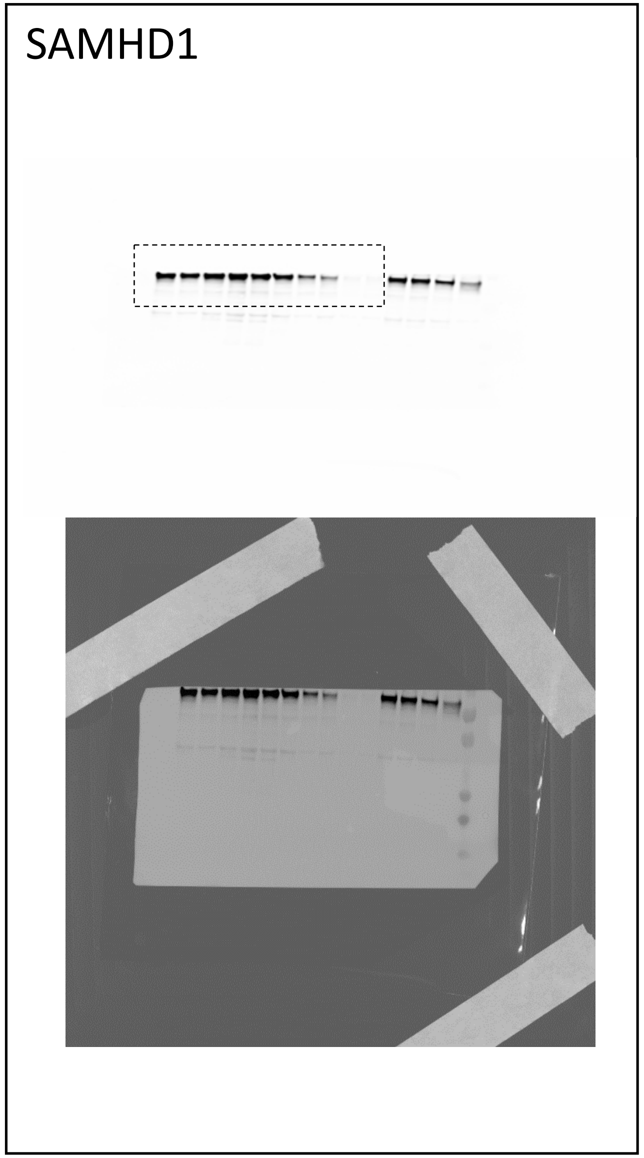

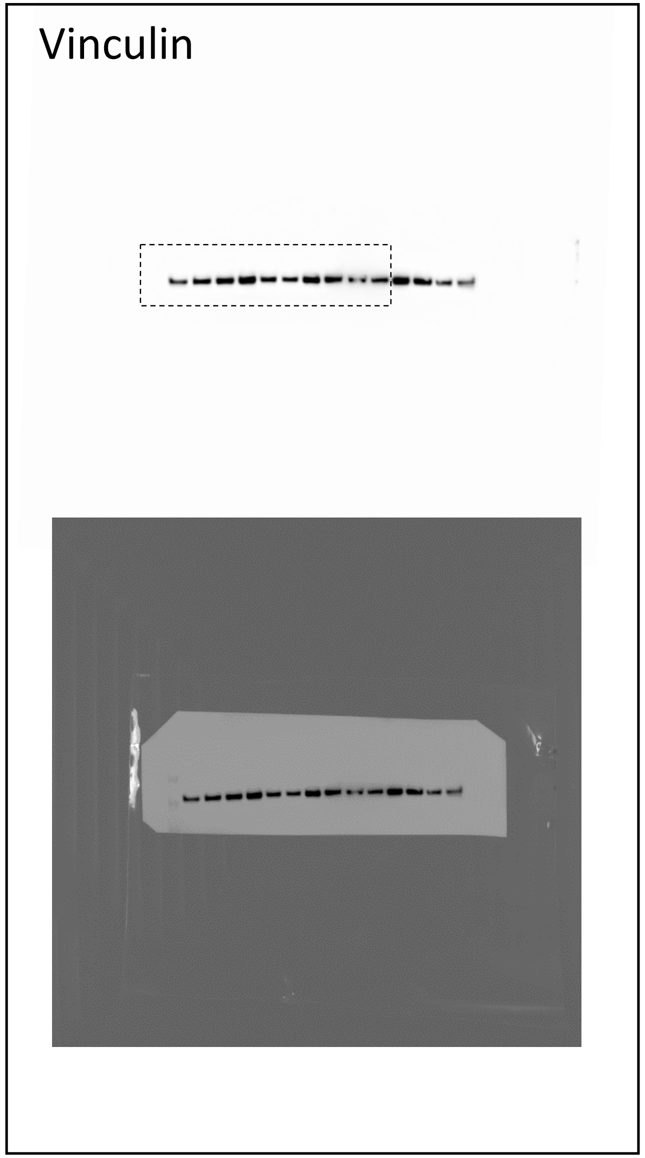

The membrane was cut and the bottom part was probed against SAMHD1, the top part against Vinculin

Source Extended Data Figure 8a

PCR SAMHD1 KI

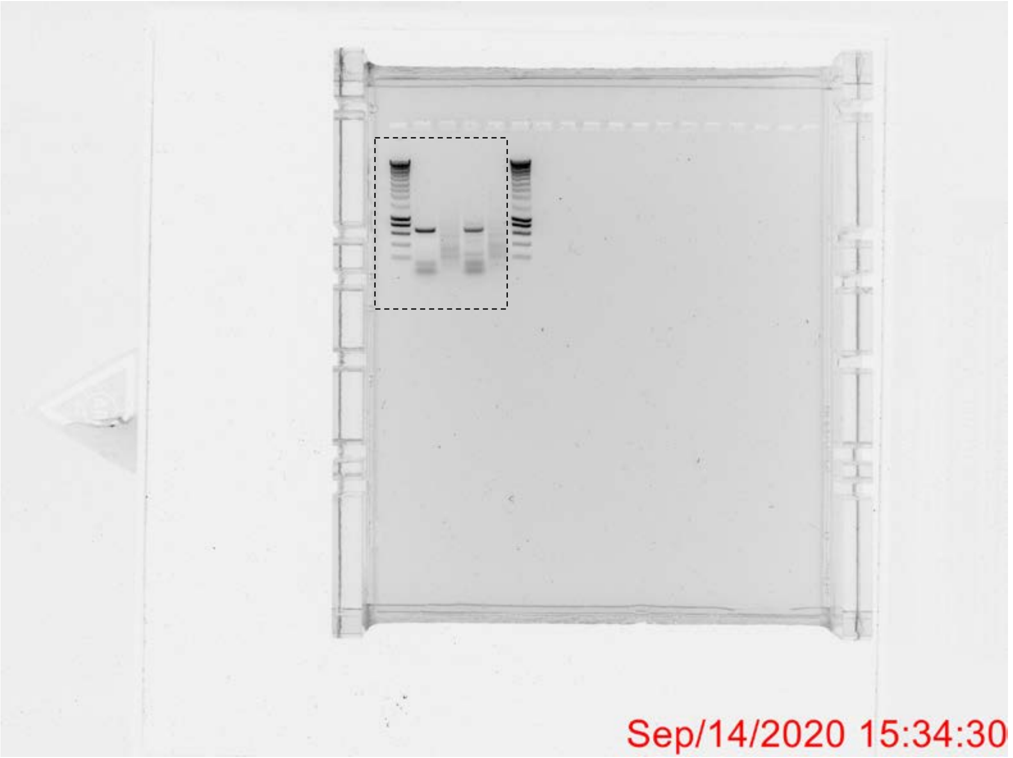

PCR *CD46 locus* (Loading control)

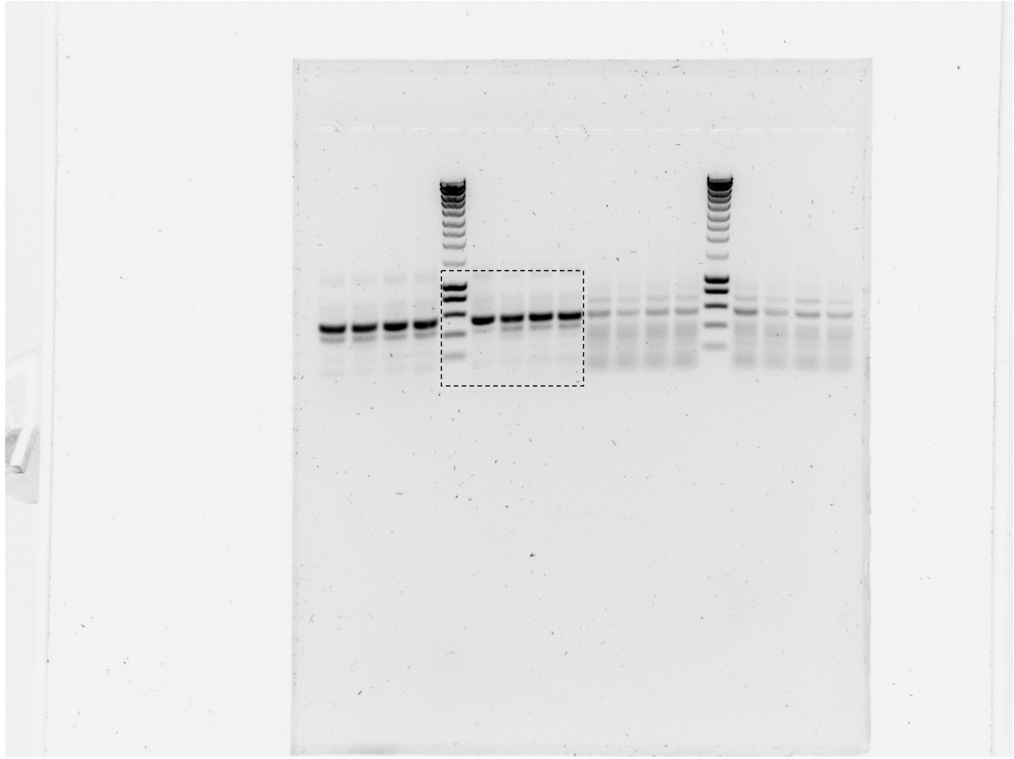

Source Extended Data Figure 9

SAMHD1

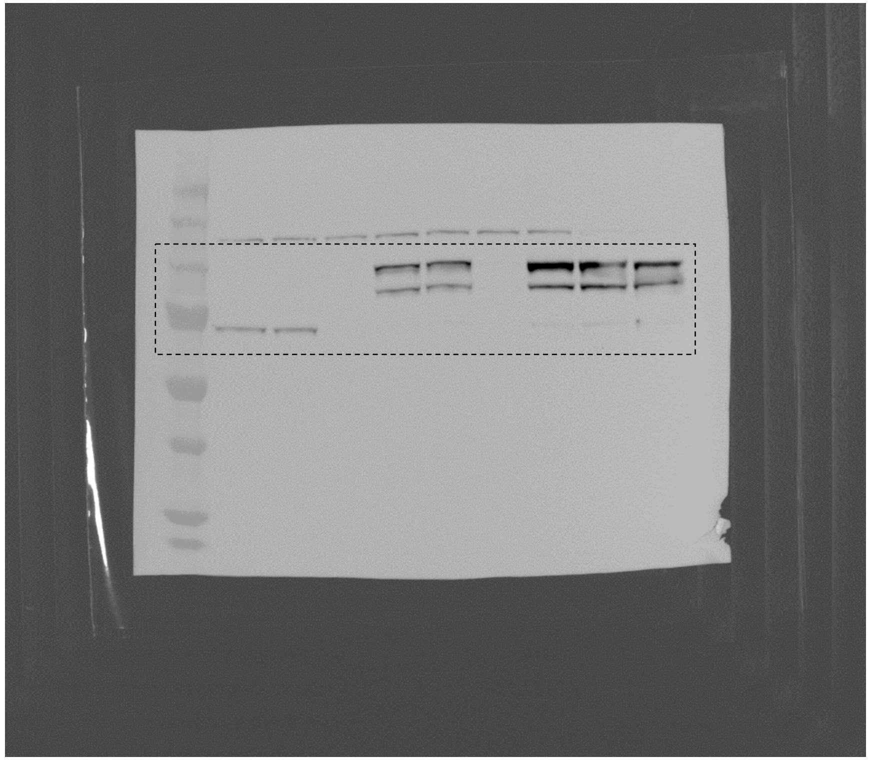

Vinculin

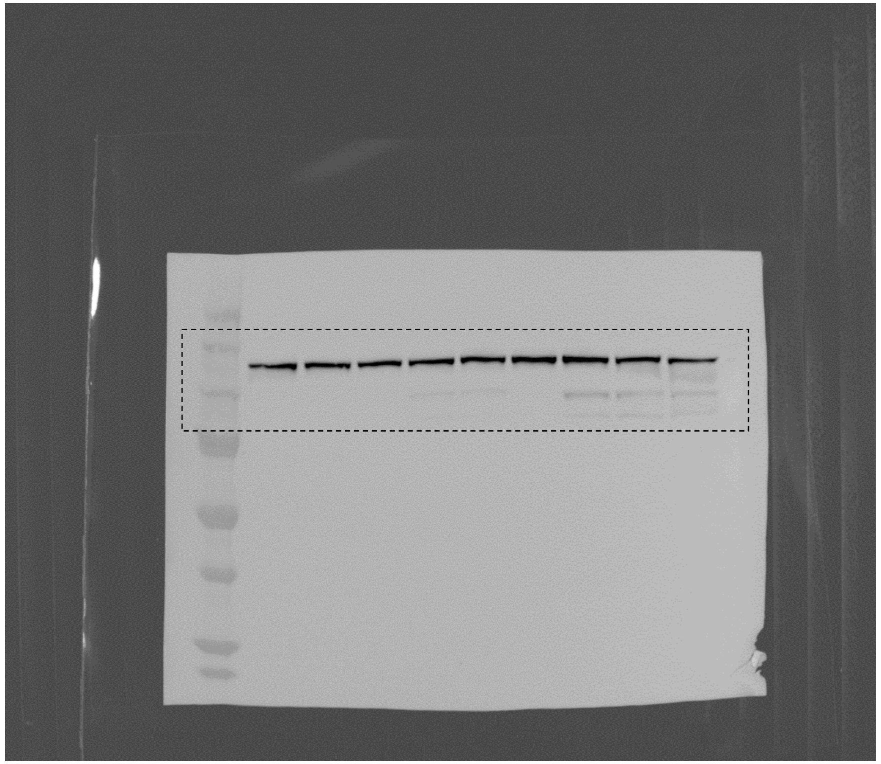

The same membrane was re-probed first against SAMHD1 and then against Vinculin.
